# Integrative co-application of citric acid and halotolerant/halophilic citrate-utilizing PGPR enhances leaf ionic homeostasis, productivity, and quality of *Vitis vinifera* L. in saline-calcareous soils

**DOI:** 10.1186/s12870-026-09150-0

**Published:** 2026-06-04

**Authors:** Asmaa G. A. Abdel Samad, Ibrahim M. Ibrahim, Hamada R. Beheiry, Ahmed Shaaban, Hamdy A. Z. Hussein

**Affiliations:** 1 Horticulture Department, Faculty of Agriculture, Fayoum University, Fayoum, 63514 Egypt; 2 Qilu University of Technology (Shandong Academy of Sciences), Shandong Analysis and Test Center, Jinan, Shandong 250014 China; 3 Department of Agricultural Microbiology, Faculty of Agriculture, Fayoum University, Fayoum, 63514 Egypt; 4 Agronomy Department, Faculty of Agriculture, Fayoum University, Fayoum, 63514 Egypt

**Keywords:** Plant-soil-microbe interaction, Organic acids, Citrate-metabolizing rhizobacteria, Biofertilization, Saline-calcareous soils, Table grape

## Abstract

**Purpose:**

Salinity severelyconstrains viticulture in calcareous soils by disrupting rhizosphere microbial community, nutrient availability, and physio-biochemical homeostasis, thereby reducing fruit yield. This study aimed to (i) assess the effects of soil-applied citric acid (CA) and halotolerant/halophilic citrate-utilizing plant growth-promoting rhizobacteria (CU-PGPR; *Bacillus spizizenii* and *Halobacillus marinus*) on the microbial community and chemical properties of saline-calcareous soil, (ii) evaluate vine physio-biochemical responses to the individual and combined treatments, and (iii) determine their impacts on yield and fruit quality.

**Methods:**

A 2-field experiment (2023/2024 and 2024/2025) on *Vitis vinifera* at the Faculty of Agriculture’s Experimental Farm (32°42′ N, 29°75′ E), Fayoum University, Egypt. This study evaluated three CA rates: 0 (CA_0_), 100 (CA_100_), and 200 (CA_200_) g vine⁻¹ season⁻¹ and three inoculation treatments: non-inoculated (NI), *H. marinus*, or *B. spizizenii*. Measurements included rhizospheric bacterial counts, soil chemistry (pH, electrical conductivity; EC_e_, and macro- and micro-nutrient availability), leaf and petiole nutrients, physio-biochemical traits, yield, and fruit quality.

**Results:**

Co-application of CA_200_ × *B. spizizenii* exhibited the strongest synergistic effects. Total bacterial count and specific functional groups markedly increased. Soil pH declined by 6.1%, while available N, K⁺, Fe²⁺, and Zn²⁺ increased by 817%, 105%, 659%, and 720%, respectively. The CA_200_ × *H. marinus* resulted in the highest available P (310% above CA_0_ × NI), though EC_e_ was unaffected. Enhanced nutrient bioavailability improved ionic balance, raising the leaf K⁺/Na⁺ ratio by 78% and the Ca²⁺/Na⁺ ratio by 74.7%, while reducing Na⁺ by 31% compared with CA_0_ × NI. Physio-biochemically, CA_200_ × *B. spizizenii* boosted vine water content, osmotic adjustment, antioxidant capacity, and photosynthetic efficiency over CA_0_ × NI. Consequently, grape yield, pruning weight, fruit TSS/acid ratio, and firmness increased by 97%, 81%, 99%, and 24%, respectively, averaged across both seasons, over CA_0_ × NI.

**Conclusion:**

Co-application of 200 g CA vine^− 1^ season⁻¹ with *B. spizizenii* inoculation effectively revitalizes microbial activity and enhances nutrient bioavailability, offering a promising strategy for sustaining viticulture under saline-calcareous soil conditions.

**Graphical Abstract:**

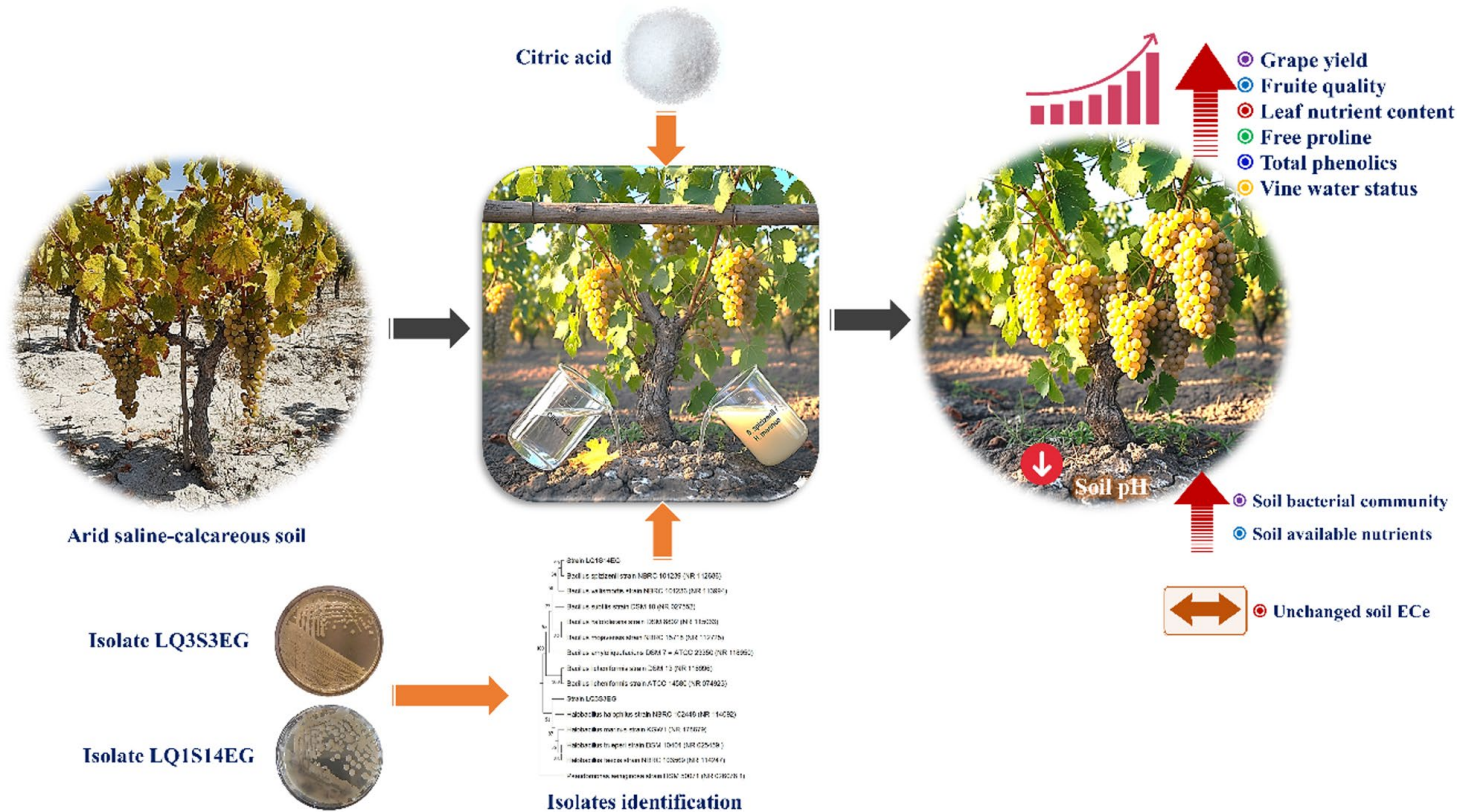

## Introduction

Table grape (*Vitis vinifera* L.), a Mediterraneanwoody vine, belongs to the Vitaceae family. It is considered one of the most economically and nutritionally important deciduous fruits widely cultivated in newly reclaimed soils, often affected by salinity and water scarcity [1]. These agroclimatic conditions are common in arid and semi-arid climates, like Egypt, which cultivates ~ 78,428 hectares of *V. vinifera*, producing ~ 1.83 million tons of berries, with an average productivity of 23.3 tons per hectare [2]. Being non-climacteric in nature and having delicate exocarp and succulent flesh, the *V. vinifera* fruits are extremely susceptible to yield and pomological traits deterioration due to osmotic/ionic stress induced by salinity and drought [3, 4]. 

Soil salinity is one of the major global agricultural challenges, considerably hindering grapevine growth, fruit yield, and its quality [5, 6]. Globally, about one-fifth of total cultivated and approximately one-third of irrigated cropland are burdened by high salinization [7]. Annually, salinization converts 1 to 2% of agriculturally productive farmland into unproductive land in arid and semi-arid zones worldwide [8, 9]. Commercial grape cultivars are regarded as moderately sensitive to soil salinity, with growth reduction commencing at 1.5 dS m^-1^ electrical conductivity for soil extract (EC_e_) or irrigation water (EC_iw_) [10] and a continual decline by ~ 9.6% for each additional dS m^-1^ unit increase [11, 12].

Once salts accumulate in the soil, excessive Na⁺ disrupts soil structural stability by promoting clay dispersion and reducing permeability, which impairs drainage, aeration, and root penetration [13–16]. These structural constraints restrict root proliferation and water uptake, exacerbating ionic imbalance and sodium/chloride (Na⁺/Cl⁻) toxicity, ultimately reducing plant water status, compromising membrane stability, and intensifying oxidative damage in sensitive fruit crops such as grapevine and mango [1, 17–19]. 

Several agricultural techniques have been adopted to mitigate salt stress, including developing field programs for breeding salt-resilient cultivars [20], grafting [21], mechanically removing surface-accumulated salts [22], and leaching salts into subsoil horizons via over-irrigation [23]. Because these techniques are time-consuming, expensive, labor-intensive, and occasionally limited in success, especially under to limited irrigation water availability in arid and semi-arid viticulture regions in Egypt, attention has shifted toward novel, integrated, cost-effective, and eco-friendly solutions. The integrative use of citric acid (CA) and citrate-utilizing plant growth-promoting rhizobacteria (CU-PGPR) offers a promising, eco-friendly approach to improve fruit tree tolerance to salinity, particularly in grapevines [24, 25].

Based on their ability to adapt to salty environments, microbiota are differentiated into halotolerant or halophilic. Halotolerant (i.e., salt-tolerant) strains can survive in media containing up to 25% NaCl or even in NaCl-free media entirely, whereas halophilic (i.e., salt-dependent) strains require salts for proliferation [26, 27]. Plant growth-promoting rhizobacteria (PGPR) offer an eco-friendly, sustainable, and viable efficient tool for supporting plant adaptation to salt-affected soil conditions and enhancing crop physiological responses, growth, and productivity [28]. As soil bio-inoculants, PGPR mitigate salinity-induced stress by root boosting of key nutrients, including nitrogen (N), phosphorus (P), potassium (K⁺), calcium (Ca²⁺), magnesium (Mg²⁺), and zinc (Zn²⁺) [29]. They also sustain ionic balance by maintaining higher K^+^/Na^+^ ratios, enhancing antioxidant defense systems, and increasing osmolyte accumulation [30, 31]. Numerous studies have confirmed that inoculation with halo-philic/tolerant PGPR significantly improves morpho-physio-biochemical attributes and yield in various field and horticultural crops subjected to saline environments, including wheat, tomato, and grapevine [32–34]. These beneficial effects are mediated through enhanced phytoregulator synthesis (e.g., indole acetic acid; IAA), 1-aminocyclopropane-1-carboxylate (ACC) deaminase (ACCD) activity, and improved rhizosphere dynamics that collectively support plant resilience against osmotic and ionic stresses [35]. PGPR can also enhance macro- and micronutrient acquisition while mitigating Na^+^ and Cl–induced nutritional perturbations under salt stress. This is partially realized by enhancing Na^+^ exclusion and upregulating the activity of high-affinity K^+^ transporters (HKTs) in root tissues, thereby maintaining a greater cellular K⁺/Na⁺ ratio [36]. This is achieved through (i) stimulating the biofilm complex formation and biopolymeric extracellular secretions (BESs), e.g., polyamines and exopolysaccharides (EPSs) on root extracellular surfaces [37, 38], which reduce Na⁺ entry, and (ii) tissue-specific downregulating of HKT1 gene expression regulating K⁺ transport [39]. Furthermore, certain PGPR stimulate plant performance by fixing atmospheric N, solubilizing soil P, K^+^, and Zn^2+^ elements, and biosynthesizing cytokinins and siderophores that enhance iron (Fe^2+^) acquisition and ACCD activity [40], thereby reducing endogenous ethylene in stressed plants.

Citric acid (CA) is a naturally occurring organic acid that plays a central role in plant energy metabolism as part of the tricarboxylic acid (Krebs) cycle [41]. When applied to soil or plants, CA can stimulate microbial activity and help reduce soil pH [42, 43], thereby improving the solubility and availability of essential nutrients [44, 45]. Its application has been shown to improve growth and physiological performance in several horticultural crops [24, 43]. Moreover, recent studies indicate that exogenous CA improves key biochemical and physiological processes that enhance plant tolerance to salinity and other environmental stresses [46, 47]. Specifically, CA has been shown to stimulate antioxidant capacity, cellular ion homeostasis, and osmotic adjustment mechanisms, together improving plant growth and productivity [48]. Soil application of CA has been demonstrated to enhance the bioavailability of essential macro- and micro-nutrients (mainly N, P, K^+^, Ca^2+^, Mg^2+^, and Zn^2+^) to maintain outstanding ion homeostasis within the rhizosphere of the salt-stressed fruit trees [49, 50].

Soil addition of CA to the salt-stressed grapevine’s rhizosphere not only enhances soil nutrient availability but also provides a readily metabolizable carbon (C) that directly supports the functionality of CU-PGPR strains and indirectly the broader rhizospheric microbiome [42, 51]. When CA is added to the rhizosphere with CA-metabolizing PGPR inoculation, these synergistic mechanisms work together to improve grapevine salt resilience by promoting rhizospheric biofilm formation, optimizing nutrient uptake, and stimulating root proliferation via the beneficial extracellular secretions.

The individual application of CA or CU-PGPR may have been reported in annual agronomic and horticultural crops for their roles in ameliorating salt-induced stress on tomato [46], eggplant [52], and soybean [53]. However, according to existing literature, no scientific report has yet explored the combined effect of rhizospheric-applied CA in conjunction with the halo-philic/tolerant CU-PGPR inoculation on perennial fruit crops, including grapevine grown under saline calcareous soil conditions. Therefore, this research aimed to underline the potential of CU-PGPR strains (*Halobacillus marinus*, a halophilic strain, and *Bacillus spizizenii*, a halotolerant strain) integrated with soil-applied CA to chemically modify the rhizosphere soil and enhance the bacterial community and antioxidant defenses. These effects are expected to improve the physio-biochemical responses, productivity, and pomological traits of table grapes under saline calcareous soil conditions. This study hypothesizes that combining rhizospheric CA application and halo-philic/tolerant CU-PGPR rhizospheric inoculation would synergistically enhance salt-stressed table grape (cv. Superior Seedless) performance by improving soil microbial activity, nutrient availability, vine physiological resilience, and yield and fruit quality.

## Materials and methods

### Plant material, study site conditions, and soil characteristics

This study was carried out on *V. vinifera* L. cv. ‘Superior Seedless’ a commercially important table grape, at the Experimental Farm in the Demo region (32^◦^ 42ˊ N, 29^◦^ 75ˊ E, and 32 m a.s.l.), Faculty of Agriculture, Fayoum University, Fayoum Governorate, Egypt. Two field trials were conducted during the 2023/2024 and 2024/2025 growing seasons. At trial initiation, the grapevines, aged five years, were uniformly in canopy size, spaced in regular rows at 3 m inter-rows and 2 m within intra-rows, equating to 1666 vines ha^-1^, and grafted onto the ‘Freedom’ rootstock, recognized for its vigorous growth and root-knot nematode resistance. Although the region is classified as arid under the Köppen–Geiger system, its agricultural production cycle displays semi-arid Mediterranean conditions in winter, as indicated by climatic records from the 2023/2024 and 2024/2025 seasons. The daytime air temperatures ranged from 20.3°C in January to 40.2°C in June, with nighttime temperatures notably lower. Relative humidity dropped from 69.4% in winter to 36.7% in summer, while wind speed remained moderate (2.2-4.0 m s⁻¹) and rainfall negligible (< 1.9 mm month⁻¹), typifying arid climate conditions.

A composite rhizospheric soil sample was collected from two layers (i.e., 0–0.2 and 0.2–0.4 m depths), using a 5-cm-diameter soil sampler, at the beginning of the trial in each season. The experimental soil’s physico-chemical properties and selected irrigation water quality before and after the two-season (2023/2024 and 2024/2025) field experiment are presented in Table 1. Baseline soil physico-chemical properties were measured once, before the 2023/2024 season, to characterize the initial field conditions. No additional pre-season sampling was performed before the 2024/2025 season; instead, soil status at the end of the 2023/2024 season served as the starting condition for the subsequent season. As per the USDA soil texture taxonomy triangle, the experimental soil was classified as sandy loam, indicating moderate hydraulic and aeration properties. Soil physico-chemical properties were determined following the standard methodologies reported by Klute and Dirksen [54] and Page et al. [55]. Specifically, soil texture (i.e., particle size distribution) of air-dried samples was determined using the hydrometer procedure, whereas dry bulk density (DBD; g cm⁻³) was measured by the core (cylinder) sampling procedure. Readily available water (RAW; %), denoting the fraction of soil moisture extractable by roots, was calculated by the subtraction of soil water at permanent wilting point (PWP; %) from soil water at field capacity (FC; %), determined at − 0.33 bar using the tension table procedure; i.e., RAW = FC − PWP. Soil pH was determined directly in a saturated soil paste at the standard soil-to-deionized water ratio (1:2.5 *w/v*) using a calibrated pH meter, and soil EC_e_ (dS m^-1^) was measured in the soil paste extract using a CM25 conductivity meter. The total CaCO₃ content was quantified by measuring the CO_2_ gas volume released upon acidification of CO₃-bearing samples using the calcimeter volumetric procedure.

**Table 1:** Initial physico-chemical properties (*n* = 3) of the rhizospheric soil (across all treatments) of *Vitis vinifera* L. cv. ‘Superior Seedless’ vines at two depths within the 0.0-0.4 m soil profile, measured before the two-season (2023/2024 and 2024/2025) field experiment

Feature	Unite	Value	
		Soil depth (m)	
		0.0-0.2	0.2–0.4
Sand	(%)	61.7	58.3
Silt		23.8	28.1
Clay		14.5	13.6
Textural category		Sandy loam	Sandy loam
Bulk density (dry basis)	(g cm^− 3^)	1.52	1.45
Moisture content at			
Field capacity	(%)	19.52	15.10
Permanent wilting point		11.69	8.63
Readily available water		8.23	6.47
pH (1: 2.5 soil: H_2_O, *w: v*)		7.60	7.56
EC_e_ (1: 2.5, *w: v*) soil extract	(dS m^− 1^)	5.25	5.82
CaCO_3_	(%)	9.92	12.2
Organic matter		0.85	0.81
^**^Cations			
Na^+^	(meq L^− 1^)	16.5	13.5
K^+^		1.12	0.87
Ca^2+^		18.64	25.3
Mg^2+^		16.2	18.5
^**^Anions			
Cl^−^	(meq L^− 1^)	26	24.2
SO_4_^2−^		22.14	28.86
HCO_3_^−^		4.32	5.11
CO_3_^2−^		---	---

^**^ for soil analysis were carried out using soil paste leachate (1:2.5 soil: H₂O, *w/v*). EC_e_= saturated soil paste extract electrical conductivity

### Citrate-utilizing plant growth-promoting rhizobacteria (CU-PGPR)

**Fig. 1:**
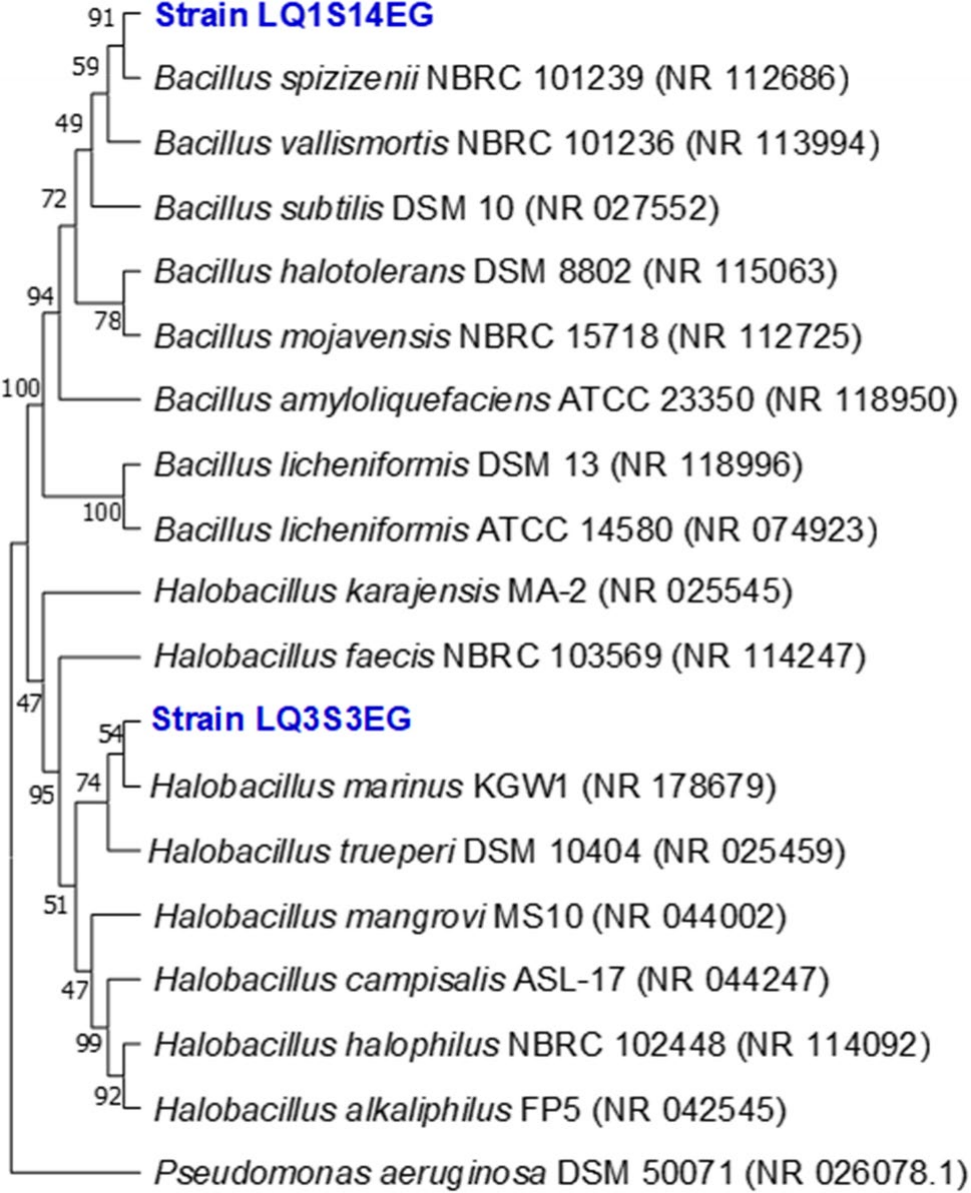
Neighbor joining tree illustrating the phylogenetic position of isolates LQ1S14EG and LQ3S3EG (marked in blue) and their closest related members. Accession numbers are given in parentheses. Bootstrap values are shown as percentages of 100 replicates

From the rhizosphere of olive trees that grow on saline soil on the shore of Lake Qarun (29.433111 N and 30.440253 E) in the Fayoum region of Egypt, halophilic bacteria (LQ3S3EG isolate) and halotolerant bacteria (LQ1S14EG isolate) were isolated using SG medium [56], supplemented with 5% NaCl. The isolates LQ3S3EG and LQ1S14EG were identified as *Halobacillus marinus* and *Bacillus spizizenii* (Fig. 1), respectively, based on their 16 S rRNA gene sequence using pair universal primers fD1; AGAGTTTGATCCTGGCTCAG and rD1; AAGGAGGTGATCCAGCC [57]. Additionally, certain functional potentials of the isolated CU-PGPR strains were evaluated, and their corresponding characteristics are presented in Table 2.

**Table 2:** Plant growth promotion properties of halophilic (strain LQ3S3EG) and halotolerant (strain LQ1S14EG) bacteria used in the present study during the 2023/2024 and 2024/2025 experimental seasons

Property	Method’s reference	Bacterial strain	
		LQ3S3EG	LQ1S14EG
Indole acetic acid production	Loper and Schroth’s [58]	Positive	Negative
Phosphate dissolving	Rodriguez et al. [59]	Positive	Positive
Zinc dissolving	Saravanan et al. [60]	Negative	Positive
Siderophores production	Schwyn and Neilands [61]	Positive	Positive
Growth on free N medium	Reis et al. [62]	Negative	Positive
Citrate utilization	MacFaddin [63]	Positive	Positive
Polymers production	Ibrahim et al. [57]^(1)^ and Zeng et al. [64]^(2)^	Exopolysaccharides^(1)^	Polyglutamine^(2)^

LQ3S3EG and LQ1S14EG bacterial strains correspond to *Halobacillus marinus* and *Bacillus spizizenii*, respectively

*N* nitrogen

### Experimental design, treatment applications, and horticultural management

For experimental rigor and statistical validity, a split-plot layout embedded in a randomized complete block design (RCBD) was implemented, allowing a dependable assessment of both main and interaction effects under field conditions. Each treatment replicated three times with two grapevines per replicate, resulting in a total of 54 experimental grapevines. The main plots were randomly assigned to the soil CA application rates, e.g., 0 as a non-CA-treated control, 100 (CA_100_), and 200 (CA_200_) g vine⁻¹ season⁻¹. The sub-plots were allocated to the rhizosphere inoculation with CU-PGPR strains, including a non-CU-PGPR-inoculated control (NI), inoculation with *H. marinus* (halophilic strain), or *B. spizizenii* (halotolerant strain). The CA was thoroughly mixed with an appropriate amount (~ 2 kg vine⁻¹) of clear fine sand and uniformly incorporated into the soil around the vine trunk at a 0.4–0.6 m radial distance in the active rooting zone. Each of the *H. marinus* or *B. spizizenii* strain inoculants (1–2 × 10⁶ cfu ml⁻¹) was inoculated into the rhizosphere soil at a distance of 0.4–0.6 m from the vine trunk, at a rate of 8 L vine⁻¹ season⁻¹, divided into four applications (each 2 L vine⁻¹). These microbial inoculations were temporally synchronized with each soil-applied CA rate, which was also applied in four equal portions: the first at late March, followed by three subsequent applications at regular monthly intervals.

In the 1st week of January of each season, grapevines were trellised using the Spanish Parron technique and winter-pruned to retain 72 buds per vine (distributed across six fruiting canes, each bearing 12 buds), aiming to optimize canopy structure and fruit-bearing capacity. During each experimental season, grapevines were minerally fertilized with 150 g of calcium superphosphate (15.5% P_2_O_5_) in early January. Additionally, 500 g of potassium sulfate (48% K_2_O) and 750 g of ammonium sulfate (20.5% N) were applied in three equal splits, commencing in mid-March onward, at one-month intervals, to support continuous nutrient supply throughout the early to mid-development stages [65]. Irrigation water was applied via a conventional open-field flood surface method using normal low-salinity irrigation water (EC_iw_= 0.57 dS m⁻¹), reflecting the standard practice in the 5-year-old vineyard orchard. To maintain uniform soil moisture and salinity conditions across plots, the orchard field was laser-leveled before the experiment, and irrigation was conducted within well-defined borders that ensured even sheet flow. A controlled gentle slope (≤ 0.2%) and lateral drainage furrows facilitated uniform advance and recession of water, preventing local ponding or uneven infiltration. Irrigation scheduling followed crop evapotranspiration (ETc), calculated from Class A pan evaporation and adjusted using real-time agro-meteorological data [66].

### Field sampling and laboratory analysis

#### Microbiological analysis of soil

At the end of the 2-year field trial, microbiological assessments of the treated soil samples were performed using culture-dependent methods. The enumeration of total bacteria count (TBC), total halophilic and halotolerant bacteria (TH-Ph/To-B), phosphate-solubilizing bacteria (PSB), and zinc-solubilizing bacteria (ZSB) was conducted via the standard plate count technique and expressed as colony-forming units (cfu) per gram of oven-dried soil. In contrast, the population of citrate-utilizing bacteria (CUB) was determined using the most probable number (MPN) method.

Selective media and incubation conditions were applied for each microbial group as follows: nutrient agar (NA) was used for TBC and TH-Ph/To-B, the latter supplemented with 5% NaCl; phosphate-solubilizing bacteria were cultured on phosphate solubilization agar (PSA) medium [59]; zinc-solubilizing bacteria on zinc solubilization agar (ZSA) medium [60]; and citrate-utilizing bacteria in Simmons citrate broth (SCB) medium [63]. All cultures were incubated at 30 °C for 2–3 days.

Soil samples were collected post-harvest, with three replicate samples obtained from separate vines for each treatment. Samples were transported in sterile plastic containers. Serial ten-fold dilutions (10^− 1^ to 10^− 9^) were prepared, and 100 µL from each dilution was inoculated onto the appropriate medium using the spread plate method, followed by incubation under defined conditions. For CUB enumeration, five replicate tubes containing Simmons’s citrate broth (SCB) medium were inoculated with 1 mL from each dilution and incubated at 30 °C for 2–3 days, in accordance with the most probable number protocol.

#### Determination of rhizospheric available nutrients, soil pH, and electrical conductivity

At the end of each experimental season, representative rhizospheric soil samples were collected from the trees of each CA × CU-PGPR treatment at two depths (0.0–0.2 and 0.2–0.4 m) to determine the available N, P, K^+^, Fe^2+^, and Zn^2+^. The results are presented as mg kg⁻¹ of dry soil, after adjustment for extract and dilution volumes and the initial soil dry weight as follows:

The available N in soil was estimated following the hot-water-extractable N procedure as originally described by Ghani et al. [67] and adapted by Curtin et al. [68]. A 10 g sample of air-dried soil (< 2 mm) was shaken/extracted with 100 mL of deionized water (soil: water ratio 1:10, *w/v*) and then incubated at 80 °C for 16 h. After cooling, extracts were centrifuged (3500 × g, 20 min) and filtered through Whatman # 42. The total N in the extract was then determined by micro-Kjeldahl analysis, involving a distillation step to convert the extracted N to NH₄⁺-N, followed by titration with a standard H_2_SO_4_ solution.

The standard colorimetric method for determining available P in soil extracted with a 0.5 M NaHCO₃ solution adjusted to a pH of 8.5 [69]. The extract is then filtered to remove soil particles. A color-developing reagent, typically containing ammonium molybdate and antimony potassium tartrate, is added to the filtrate. Ascorbic acid, as a reducing agent, was then added to reduce the complex to molybdenum blue. The absorbance of the resulting solution is measured at 880 nm using a UV–Vis spectrophotometer, and the P concentration (mg P L^− 1^) is calculated by comparing the absorbance to a standard calibration curve.

The available K⁺, Fe^+ 2^, and Zn^2+^ nutrients were determined using the 1.0 M NH₄OAc extraction method buffered at pH 7.0 [70]. Briefly, 2 g of air-dried, sieved soil was shaken with 20 mL of the extraction solution for 1/2 h. The resulting suspension was filtered, and the concentration of K⁺, Fe^+ 2^, and Zn^2+^ nutrients in the clear filtrate was quantified using a Spectra-55 AA flame atomic absorption spectrometer (Agilent Technologies, CA, USA) with an air–acetylene flame. The instrument was calibrated with a series of standards prepared in the same 1.0 M NH₄OAc solution. Soil pH was determined directly in a saturated soil paste at the standard soil-to-deionized water ratio (1:2.5 *w/v*) using a calibrated pH meter, and soil ECe (dS m^− 1^) was measured in the soil paste extract using a CM25 conductivity meter.

#### Physio-biochemical responses

At the end of July in each growing season, twenty fully expanded and physiologically mature grapevine leaves were randomly sampled to determine key plant physio-biochemical responses indicative of functional status. For vine water status, the MSI was assessed following the method of Premchandra et al. [71] with minor modifications by Sairam [72]. In brief, fully expanded leaf discs were excised and immersed in 10 mL of double-distilled water (DdW) in a thermostatically controlled water bath at 40 °C for 1/2 h. The initial electrical conductivity (IEC) of the bathing solution was then measured using a conductivity meter. Posteriorly, the same samples were boiled at 100 °C for 10 min to get the final electrical conductivity (FEC). The MSI was calculated as per the formula MSI$= \left[1-\left(\mathrm{IEC/FEC}\right)\right]\times100$. The vine relative water content (VRWC) of fully expanded grapevine leaves was determined according to the Barrs and Weatherley’s [73] procedure. Fresh weight (FrW) was immediately recorded after leaf detachment. Thereafter, leaf samples were submerged in glass tubes filled with DdW and incubated at 4 °C for 24 h to get their saturated weight (StW). Subsequently, samples were oven-dried at 80 °C for 24 h to determine their dry weight (DrW). The VRWC was then calculated using the following equation:$\mathrm{VRWC}\left(\%\right)=\left[\left(\mathrm{FrW-DrW}\right)/\left(\mathrm{StW-DrW}\right)\right]\times100$.

To assess osmoregulatory (e.g., free proline expressed as µg g⁻¹ DrW) and non-enzymatic (e.g., total phenolic (TPh) content expressed as mg g⁻¹ DrW) compounds, free proline content (ProC) was extracted using 3% sulfosalicylic acid and colorimetrically quantified with acid ninhydrin reagent, following Bates et al. [74]. The TPh content was spectrophotometrically measured using the Folin–Ciocalteu reagent, as outlined by Ainsworth and Gillespie’s [75] technique.

#### Macro-and micronutrient contents and ionic homeostasis in leaves and petioles

Representative typical foliage samples, comprising 25 fully expanded mature leaves located opposite the 1st fruit cluster on the current season’s canes per vine as per Demirer et al. [76], were picked during the 3rd week of August in each season for nutrient determination. The samples were dried in a forced-air oven at 70 °C until constant weight, finely ground in a stainless-steel grinder, and kept in airtight polyethylene pouches in moisture-free conditions for later macro (N, P, K^+^, Ca^2+^, and Na^+^ as %)- and micro-nutrient (Fe^2+^, Zn^2+^, and Mn^2+^ as mg kg^− 1^ DrW) determination. The N content was colorimetrically determined using the Orange G dye reagent as described by Hafez and Mikkelsen [77], which facilitates accurate determination of total N concentrations in grapevine leaf tissue. The P content was spectrophotometrically determined based on the development of a blue complex between phosphate ions and ammonium molybdate in an acidic condition, as described by Jackson’s [78] procedure. The K^+^, Na^+^, Ca^2+^, Fe²^+^, Zn^2+^, and Mn²⁺ in grapevine leaves and petioles were determined using a Spectra-55 AA flame atomic absorption spectrometer (Agilent Technologies, CA, USA) following the protocol established by Johnson and Ulrich [79], then the ratios of K^+^/Na^+^ and Ca^2+^/Na^+^ were calculated.

#### Pruning wood weight, grape yield, and quality traits

Pruning wood weight (WW), as a key indicator of vine vigor, was expressed as kg of fresh weight per vine (kg vine^− 1^) during the winter pruning periods in January 2024 and 2025. Immediately following commercial hand pruning, the fresh pruned biomass of each vine was individually field-weighed. Fresh pruning samples (500 g) were taken and dried in a forced-air oven at 70 °C to a constant weight to determine the dry matter percentage (DM%). Posteriorly, the pruning DrW per vine, as an indicator of seasonal biomass allocation, was calculated by multiplying pruning DM% by fresh weight per vine (kg vine^− 1^). At the marketable stage, the number of clusters per vine was counted. Subsequently, five clusters were randomly sampled from each vine to determine the average cluster weight. The total yield in kg vine^− 1^ was calculated and then extrapolated to field-scale productivity (t ha^− 1^) based on planting configuration. A 25-fresh-fruit sample from each replication was randomly taken to determine average fruit firmness. Flesh firmness was measured using a fruit firmness digital penetrometer (Model FT 327, Milan, Italy) at two opposite sides of each fruit, and the readings were expressed in kg cm^− 2^. Following the AOAC [80] guidelines, the total soluble solids (TSS%), expressed in °Brix, in freshly extracted juice at 25 °C, were measured using a digital calibrated hand-held refractometer (Carl Zeiss Jena, Germany). Juice total acidity (AC%) was determined as tartaric acid equivalents and quantified titrimetrically according to AOAC [80] through neutralization with 0.1 N NaOH and phenolphthalein as a pH visual indicator until a faint light pink endpoint.

### Statistical analysis

Prior to conducting analysis of variance (ANOVA) on each variable, the Shapiro–Wilk test (*p* > 0.05) was applied to verify data normality in line with the assumptions outlined by Gomez and Gomez [81]. Data were subjected to analysis using the General Linear Model procedure in INFOSTAT computer software (v.2020 statistical package, Córdoba University, Córdoba, Argentina; [82] under a split-plot in an RCBD layout. Post hoc comparisons of treatment means were performed using Duncan’s test at a significance level of ≤ 0.05.

## Results

### Soil rhizobacterial communities

The results clearly demonstrate that increasing CA soil application rates, inoculation with CU-PGPR strains, or their interaction significantly (*p* < 0.0001–0.0018; Table 3) enhanced all counted rhizospheric bacterial communities compared to their controls. The individual application of CA as an organic acid amendment or inoculation with CU-PGPR strains significantly influenced all assessed rhizobacterial groups. Increasing the CA rate from 0 to 200 g vine^− 1^ led to a progressive and significant increment in TBC (0.45 to 6.54 × 10^10^ cfu g^− 1^ dry soil), H-Ph/Tol-B count (0.11 to 2.89 × 10^9^ cfu g^− 1^ dry soil), CUB count (0.33 to 5.35 × 10^8^ cfu g^− 1^ dry soil), PSB count (0.52 to 3.24 × 10^7^ cfu g^− 1^ dry soil), and ZnSB count (0.08 to 0.39 × 10^7^ cfu g^− 1^ dry soil), all peaking significantly at CA_200_. Similarly, inoculation stressed grapevines with CU-PGPR strains, particularly *B. spizizenii* strain, markedly increased all counted bacterial groups in their root and rhizospheric zones compared to the NI-treated control trees, with maximizing bacterial count for TBC (5.99 × 10¹⁰ cfu g^− 1^ dry soil), H-Ph/Tol-B count (3.14 × 10^9^ cfu g^− 1^ dry soil), CUB count (5.38 × 10^8^ cfu g^− 1^ dry soil), PSB count (4.30 × 10^7^ cfu g^− 1^ dry soil), and ZnSB count (0.58 × 10^7^ cfu g^− 1^ dry soil). In contrast, the lowest counts for all rhizobacterial groups were consistently recorded under the CA_0_ or non-inoculated treatment as an individual effect.

**Table 3:** Effect of citric acid (CA) soil application rate, inoculation with citrate-utilizing plant growth-promoting rhizobacteria (CU-PGPR) strains, and their interaction on rhizospheric bacterial counts of *Vitis vinifera* L. cv. ‘Superior Seedless’ under arid saline calcareous soil conditions, evaluated at the end of the two-year (2023/2024 and 2024/2025 seasons) field experiment

Factor		TBC (cfu×10^10^ g^− 1^ dry soil)	TH-Ph/Tol-B (cfu×10^9^ g^− 1^ dry soil)	CUB (cfu×10^8^ g^− 1^ dry soil)	PSB (cfu×10^7^ g^− 1^ dry soil)	ZnSB (cfu×10^7^ g^− 1^ dry soil)
CA						
CA_0_		0.45 ± 0.10c	0.11 ± 0.03c	0.33 ± 0.07c	0.52 ± 0.140c	0.08 ± 0.010c
CA_100_		3.29 ± 0.55b	0.77 ± 0.26b	1.59 ± 0.44b	1.88 ± 0.580b	0.19 ± 0.080b
CA_200_		6.54 ± 0.94a	2.89 ± 0.66a	5.35 ± 0.94a	3.24 ± 0.830a	0.39 ± 0.070a
CU-PGPR						
NI		1.06 ± 0.27c	0.05 ± 0.01c	0.34 ± 0.07c	0.01 ± 0.002c	0.03 ± 0.007b
* H. marinus*		3.22 ± 0.91b	0.58 ± 0.15b	1.54 ± 0.43b	1.33 ± 0.23b	0.04 ± 0.006b
* B. spizizenii*		5.99 ± 0.81a	3.14 ± 0.72a	5.38 ± 0.84a	4.30 ± 0.98a	0.58 ± 0.026a
CA × CU-PGPR						
CA_0_	NI	0.11 ± 0.01e	0.02 ± 0.002d	0.09 ± 0.01c	0.002 ± 0.0002e	0.01 ± 0.001d
	*H. marinus*	0.46 ± 0.04e	0.09 ± 0.005d	0.33 ± 0.02c	0.59 ± 0.052de	0.02 ± 0.001d
	*B. spizizenii*	0.77 ± 0.04e	0.20 ± 0.008d	0.56 ± 0.04c	0.98 ± 0.058de	0.20 ± 0.006c
CA_100_	NI	1.12 ± 0.10de	0.05 ± 0.004d	0.38 ± 0.03c	0.01 ± 0.001e	0.04 ± 0.003d
	*H. marinus*	2.65 ± 0.24c	0.52 ± 0.032d	1.12 ± 0.12c	1.25 ± 0.079d	0.04 ± 0.004d
	*B. spizizenii*	6.11 ± 0.42b	1.76 ± 0.151b	3.26 ± 0.19b	4.38 ± 0.679b	0.49 ± 0.041b
CA_200_	NI	1.96 ± 0.14 cd	0.09 ± 0.004d	0.55 ± 0.04c	0.02 ± 0.003e	0.05 ± 0.006d
	*H. marinus*	6.57 ± 0.60b	1.12 ± 0.081c	3.18 ± 0.26b	2.16 ± 0.092c	0.06 ± 0.006d
	*B. spizizenii*	11.10 ± 0.56a	7.47 ± 0.561a	12.33 ± 1.53a	7.53 ± 0.524a	1.05 ± 0.046a
Significance						
CA		< 0.0001^**^	0.0003^**^	0.0006^**^	0.0018^**^	< 0.0001^**^
CU-PGPR		< 0.0001^**^	< 0.0001^**^	< 0.0001^**^	< 0.0001^**^	< 0.0001^**^
CA × CU-PGPR		< 0.0001^**^	< 0.0001^**^	< 0.0001^**^	< 0.0001^**^	< 0.0001^**^

Values are presented as means ± standard error (*n* = 3). Means followed by the same letter are not significantly different at *p* > 0.05 according to Duncan’s multiple range test. Asterisks denote statistical significance (^**^p ≤ 0.01). cfu=colony forming unit

*CA*_0_ non-CA-treated vines, *CA*_100_ CA at 100 g vine^− 1^, *CA*_200_ CA at 200 g vine^− 1^, *NI* non-CU-PGPR-inoculated vines, *H. marinus*= inoculation with LQ3S3EG isolate, *B. spizizenii*= inoculation with LQ1S14EG isolate, *TBC* Total bacterial count, *H-Ph/Tol-B* Total halo-philic/tolerant bacteria, *CUB* Citrate-utilizing bacteria, *PSB* Phosphate-solubilizing bacteria, *ZnSB* Zinc-solubilizing bacteria

For the interaction of CA rate × CU-PGPR strain, the highest counts for TBC, H-Ph/Tol-B, CUB, PSB, and ZnSB were consistently recorded under the combined application of CA_200_ with *B. spizizenii* strain inoculation, reaching 11.10 × 10^10^, 7.47 × 10^9^, 12.33 × 10^8^, 7.53 × 10^7^, and 1.05 × 10^7^ cfu g^− 1^ dry soil, respectively. In contrast, the lowest bacterial counts were observed in the CA_0_ × NI-treated control trees, particularly for PSB (0.002 × 10^7^ cfu g^− 1^ dry soil) and ZnSB (0.01 × 10^7^ cfu g^− 1^ dry soil).

### Changes in rhizospheric nutrient availability, soil pH, and electrical conductivity

Across both seasons, rhizospheric CA soil application, CU-PGPR inoculation, and their interaction markedly affected the rhizospheric N, P, K⁺, Fe²⁺, and Zn²⁺ availability in both 2023/2024 and 2024/2025 seasons (Tables 4 and 5). Regarding the CA effect, raising the rate from CA_0_ to CA_200_ significantly increased Av. N by 363 and 309%, Av. P by 40 and 38%, and Av. K⁺ by 14 and 14%, alongside sharp rises in Av. Fe²⁺ (73 and 65%) and Av. Zn²⁺ (287 and 213%), respectively. With CU-PGPR, inoculation with *B. spizizenii* was consistently superior, enhancing Av. N by 287 and 238%, Av. P by 63 and 61%, Av. K⁺ by 77 and 76%, Av. Fe²⁺ by 268 and 180%, and Av. Zn²⁺ by 330 and 230% compared with NI across both years, followed by *H. marinus* with moderate gains. The CA × CU-PGPR interaction exhibited the most pronounced synergistic improvements, particularly CA_200_ × *B. spizizenii*, which maximized nutrient availability (Av. N by 913 and 721%, Av. K⁺ by 106 and 104%, Av. Fe²⁺ by 810 and 507%, and Av. Zn²⁺ by 804 and 635%), while CA_200_ × *H. marinus* achieved the greatest P availability (Av. P by 320 and 299%) compared with CA_0_ × NI in 2023/2024 and 2024/2025, respectively.

**Table 4:** Effect of citric acid (CA) soil application rate, inoculation with citrate-utilizing plant growth-promoting rhizobacteria (CU-PGPR) strains, and their interaction on available (Av.) nitrogen (N), phosphorus (P), and potassium (K^+^) in the rhizospheric soil of *Vitis vinifera* L. cv. ‘Superior Seedless’ grown under arid saline–calcareous soil conditions during the 2023/2024 and 2024/2025 seasons

Factor		Av. *N* (mg kg^− 1^ soil)		Av. *P* (mg kg^− 1^ soil)		Av. K^+^ (mg kg^− 1^ soil)	
		2023/2024	2024/2025	2023/2024	2024/2025	2023/2024	2024/2025
CA							
CA_0_		7.05 ± 0.69c	8.23 ± 0.71c	43.49 ± 6.56c	44.67 ± 6.56c	483.67 ± 42.20c	488.37 ± 42.06c
CA_100_		9.15 ± 0.89b	10.33 ± 0.89b	45.77 ± 6.54b	46.94 ± 6.55b	528.37 ± 45.35b	530.44 ± 44.97b
CA_200_		32.64 ± 6.38a	33.64 ± 6.38a	60.66 ± 4.70a	61.66 ± 4.73a	552.52 ± 36.76a	555.85 ± 37.36a
CU-PGPR							
NI		5.86 ± 0.37c	7.03 ± 0.37c	39.32 ± 3.95c	40.50 ± 3.91c	367.03 ± 13.29c	370.43 ± 12.75c
* H. marinus*		20.34 ± 5.84b	21.39 ± 5.82b	46.51 ± 4.84b	47.56 ± 4.84b	549.00 ± 9.03b	552.14 ± 9.13b
* B. spizizenii*		22.65 ± 5.15a	23.78 ± 6.10a	64.09 ± 1.16a	65.21 ± 1.18a	648.52 ± 11.89a	652.09 ± 11.06a
CA × CU-PGPR							
CA_0_	NI	4.66 ± 0.33f	5.86 ± 0.43f	18.23 ± 0.62 g	19.43 ± 0.72 g	320.52 ± 0.87i	325.72 ± 0.96 h
	*H. marinus*	7.22 ± 0.40e	8.32 ± 0.50e	50.08 ± 0.51d	51.18 ± 0.60d	527.81 ± 1.48f	532.48 ± 1.80e
	*B. spizizenii*	9.28 ± 0.31d	10.51 ± 0.35d	62.16 ± 0.60c	63.39 ± 0.58c	602.67 ± 1.45c	606.90 ± 2.02c
CA_100_	NI	5.81 ± 0.19ef	7.01 ± 0.16ef	23.27 ± 0.64f	24.47 ± 0.58f	368.45 ± 4.43 h	372.32 ± 5.61 g
	*H. marinus*	10.14 ± 0.18 cd	11.24 ± 0.17 cd	45.49 ± 0.87e	46.59 ± 0.95e	534.33 ± 0.33e	535.43 ± 0.43e
	*B. spizizenii*	11.49 ± 0.77c	12.72 ± 0.79c	68.55 ± 0.29b	69.78 ± 0.24b	682.33 ± 0.33a	683.57 ± 0.27a
CA_200_	NI	7.10 ± 0.07e	8.23 ± 0.03e	43.96 ± 0.04e	44.93 ± 0.37e	412.11 ± 0.48 g	413.25 ± 0.57f
	*H. marinus*	43.64 ± 0.32b	44.61 ± 0.35b	76.47 ± 0.29a	77.60 ± 0.36a	584.87 ± 0.47d	588.50 ± 1.40d
	*B. spizizenii*	47.19 ± 0.61a	48.09 ± 0.91a	61.55 ± 0.87c	62.45 ± 0.60c	660.57 ± 0.30b	665.80 ± 0.81b
Significance							
CA		< 0.0001^**^	< 0.0001^**^	< 0.0001^**^	< 0.0001^**^	< 0.0001^**^	< 0.0001^**^
CU-PGPR		< 0.0001^**^	< 0.0001^**^	< 0.0001^**^	< 0.0001^**^	< 0.0001^**^	< 0.0001^**^
CA × CU-PGPR		< 0.0001^**^	< 0.0001^**^	< 0.0001^**^	< 0.0001^**^	< 0.0001^**^	< 0.0001^**^

Values are presented as means ± standard error (*n* = 3). Means followed by the same letter are not significantly different at *p* > 0.05 according to Duncan’s multiple range test. Asterisks denote statistical significance (^**^p ≤ 0.01)

*CA*_0_ non-CA-treated vines, *CA*_100_ CA at 100 g vine^− 1^, *CA*_200_ CA at 200 g vine^− 1^, *NI* non-CU-PGPR-inoculated vines, *H. marinus*= inoculation with LQ3S3EG isolate, *B. spizizenii*= inoculation with LQ1S14EG isolate

**Table 5:** Effect of citric acid (CA) soil application rate, inoculation with citrate-utilizing plant growth-promoting rhizobacteria (CU-PGPR) strains, and their interaction on available (Av.) iron (Fe^+2^), zinc (Zn^+2^), soil reaction (pH), and electrical conductivity of the saturated paste extract (ECe) in the rhizospheric soil of *Vitis vinifera* L. cv. ‘Superior Seedless’ grown under arid saline–calcareous soil conditions during the 2023/2024 and 2024/2025 seasons

Factor		Av. Fe^+ 2^ (mg kg^− 1^ soil)		Av. Zn^+ 2^ (mg kg^− 1^ soil)		Soil pH (%)		Soil EC_e_ (dS m^− 1^)	
		2023/2024	2024/2025	2023/2024	2024/2025	2023/2024	2024/2025	2023/2024	2024/2025
CA									
CA_0_		37.80 ± 12.0b	49.47 ± 10.9c	0.76 ± 0.01c	1.03 ± 0.04c	7.53 ± 0.04a	7.50 ± 0.06a	5.43 ± 0.04	5.39 ± 0.05
CA_100_		65.41 ± 13.4a	78.74 ± 13.9b	1.51 ± 0.07b	1.80 ± 0.26b	7.20 ± 0.05b	7.18 ± 0.06b	5.16 ± 0.04	5.23 ± 0.06
CA_200_		65.47 ± 14.4a	81.58 ± 15.6a	2.94 ± 0.88a	3.22 ± 0.87a	7.07 ± 0.06c	7.04 ± 0.05c	5.32 ± 0.05	5.14 ± 0.07
CU-PGPR									
NI		29.50 ± 2.8c	43.39 ± 4.0b	0.76 ± 0.02c	1.06 ± 0.05c	7.38 ± 0.05a	7.34 ± 0.07a	5.33 ± 0.05	5.30 ± 0.04
* H. marinus*		30.50 ± 5.3b	44.94 ± 5.1b	1.18 ± 0.11b	1.48 ± 0.09b	7.25 ± 0.07b	7.23 ± 0.06b	5.37 ± 0.05	5.23 ± 0.05
* B. spizizenii*		108.68 ± 5.8a	121.46 ± 7.9a	3.27 ± 0.83a	3.50 ± 0.84a	7.17 ± 0.06c	7.15 ± 0.05c	5.21 ± 0.06	5.22 ± 0.05
CA × CU-PGPR									
CA_0_	NI	13.43 ± 1.1 g	23.43 ± 2.4 g	0.71 ± 0.14 g	0.91 ± 0.06f	7.68 ± 0.04a	7.62 ± 0.05a	5.21 ± 0.04	5.20 ± 0.05
	*H. marinus*	14.37 ± 0.7 g	32.70 ± 4.5 fg	0.77 ± 0.18 fg	1.17 ± 0.06e	7.44 ± 0.04c	7.44 ± 0.04b	5.45 ± 0.07	5.47 ± 0.08
	*B. spizizenii*	85.60 ± 0.3c	92.27 ± 1.9c	0.80 ± 0.18ef	1.00 ± 0.03ef	7.47 ± 0.05b	7.45 ± 0.05b	5.62 ± 0.04	5.49 ± 0.05
CA_100_	NI	30.40 ± 0.5f	45.40 ± 5.3e	0.72 ± 0.15 g	1.09 ± 0.07ef	7.25 ± 0.05d	7.23 ± 0.05c	5.28 ± 0.05	5.31 ± 0.08
	*H. marinus*	47.70 ± 0.3d	61.03 ± 3.0d	1.25 ± 0.14d	1.51 ± 0.07d	7.21 ± 0.05e	7.19 ± 0.04d	5.25 ± 0.04	5.21 ± 0.07
	*B. spizizenii*	118.13 ± 0.7b	129.80 ± 6.6b	2.57 ± 0.18b	2.80 ± 0.13b	7.13 ± 0.04f	7.12 ± 0.03e	4.95 ± 0.05	5.17 ± 0.08
CA_200_	NI	29.43 ± 0.2f	41.10 ± 4.2ef	0.86 ± 0.15e	1.19 ± 0.09e	7.21 ± 0.04e	7.17 ± 0.04d	5.50 ± 0.06	5.41 ± 0.06
	*H. marinus*	44.76 ± 0.4e	61.33 ± 4.1d	1.53 ± 0.12c	1.77 ± 0.09c	7.09 ± 0.03 g	7.06 ± 0.05f	5.40 ± 0.05	5.01 ± 0.06
	*B. spizizenii*	122.30 ± 0.7a	142.30 ± 4.3a	6.42 ± 0.16a	6.69 ± 0.14a	6.92 ± 0.04 h	6.88 ± 0.05 g	5.05 ± 0.06	5.00 ± 0.05
Significance									
CA		< 0.0001^**^	0.0089^**^	0.0001^**^	< 0.0001^**^	< 0.0001^**^	< 0.0001^**^	0.21813^ns^	0.42321^ns^
CU-PGPR		< 0.0001^**^	< 0.0001^**^	< 0.0001^**^	< 0.0001^**^	< 0.0001^**^	< 0.0001^**^	0.5332^ns^	0.8948^ns^
CA × CU-PGPR		< 0.0001^**^	0.0002^**^	< 0.0001^**^	< 0.0001^**^	< 0.0001^**^	< 0.0001^**^	0.2005^ns^	0.5865^ns^

Values are presented as means ± standard error (*n* = 3). Means followed by the same letter are not significantly different at *p* > 0.05 according to Duncan’s multiple range test. Asterisks denote statistical significance (^**^p ≤ 0.01) and “ns” denotes a non-significant difference (*p* > 0.05)

*CA*_0_ non-CA-treated vines, *CA*_100_ CA at 100 g vine^− 1^, *CA*_200_ CA at 200 g vine^− 1^, *NI* non-CU-PGPR-inoculated vines, *H. marinus*= inoculation with LQ3S3EG isolate, *B. spizizenii*= inoculation with LQ1S14EG isolate

Soil application of CA markedly reduced the rhizospheric soil pH of *V. vinifera* L. cv. ‘Superior Seedless’ during both the 2023/2024 and 2024/2025 seasons (Table 5). Compared with the CA_0_, CA_200_ decreased soil pH by 6.1 and 6.1% during the 2023/2024 and 2024/2025 seasons, respectively. Similarly, rhizospheric inoculation with CU-PGPR significantly modulated pH, with *B. spizizenii* exhibiting the greatest impact, lowering pH by 2.8 and 2.6% relative to the NI control in the 2023/2024 and 2024/2025 seasons, respectively. The combined CA × CU-PGPR interaction revealed a synergistic reduction in soil pH, particularly under the CA_200_ × *B. spizizenii* treatment, which decreased soil pH by 9.9 and 9.7% relative to the CA_0_ × NI treatment in the 2023/2024 and 2024/2025, respectively. In contrast, soil EC_e_ remained statistically unaffected by CA rate, CU-PGPR inoculation, or their interaction in both seasons (*p* > 0.05) during the experimental period.

### Changes in macro- and micronutrient contents and ionic homeostasis in leaves and petioles

**Fig. 2:**
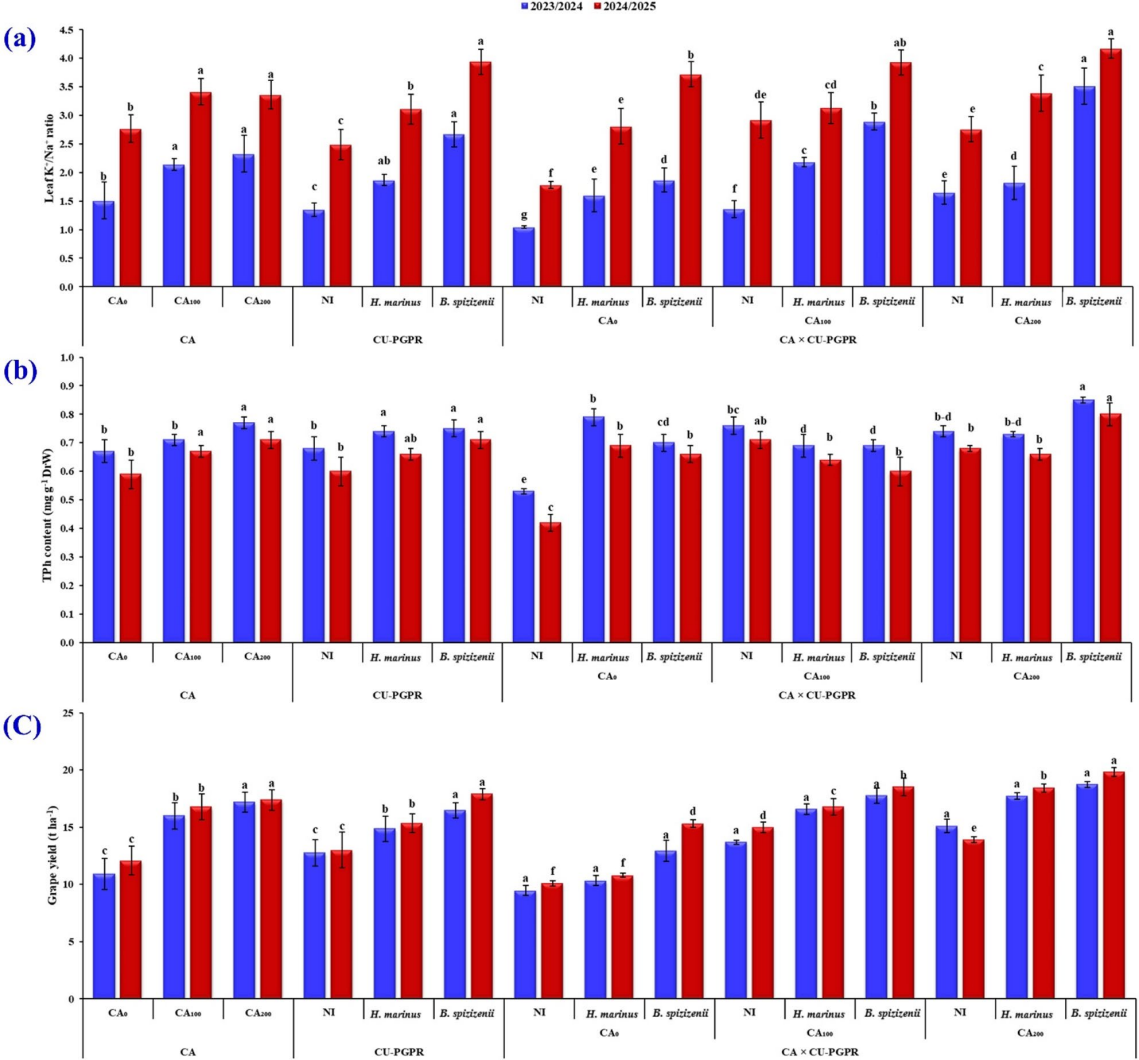
Effect of citric acid (CA) soil application rate, inoculation with citrate-utilizing plant growth-promoting rhizobacteria (CU-PGPR) strains, and their interaction on *Vitis vinifera* L. cv. ‘Superior Seedless’ grown under arid saline–calcareous soil conditions during the 2023/2024 and 2024/2025 seasons. (**a**) leaf K⁺/Na⁺ ratio, (**b**) total phenolic (TPh) content, and (**c**) grape yield in *Vitis vinifera* L. vines treated with different CA, CU-PGPR, and their CA × CU-PGPR interaction. Each vertical bar represents the mean ± standard error (*n* = 3). Different letters assigned to CA, CU-PGPR, or their interaction (CA × CU-PGPR) indicate significant differences at *p* ≤ 0.05, whereas bars sharing the same letter do not differ significantly (*p* > 0.05) according to Duncan’s multiple range test

In both seasons, CA soil application markedly enhanced leaf N, P, K⁺, Ca²⁺, and the associated K⁺/Na⁺ and Ca²⁺/Na⁺ ratios, while lowering Na⁺ accumulation (Tables 6 and 7; Fig. 2a). The most pronounced improvements were observed at CA_200_, which increased N by 20.1 and 23.7%, P by 8.3 and 16.7%, K⁺ by 29.8 and 13.6%, and Ca²⁺ by 19.7 and 27.4%, while reducing Na⁺ by 15.2 and 17.4% in 2023/2024 and 2024/2025, respectively, relative to CA_0_. These alterations led to 54.3 and 21.3% increases in the K⁺/Na⁺ ratio and 41.5 and 52.4% in the Ca²⁺/Na⁺ ratio in 2023/2024 and 2024/2025, respectively. Specifically, *H. marinus* increased leaf N by 12.2 and 6.1%, P by 17.4 and 7.7%, K⁺ by 16.7 and 4.8%, and improved the K⁺/Na⁺ ratio by 38.5 and 24.9% and Ca²⁺/Na⁺ ratio by 34.3 and 46.0% during 2023/2024 and 2024/2025, respectively, while reducing Na⁺ by 19.4 and 20.0%. Similarly, *B. spizizenii* boosted leaf N by 26.0 and 10.9%, P by 8.7 and 0.0%, K⁺ by 33.3 and 8.1%, and increased the K⁺/Na⁺ ratio by 97.8 and 58.2% and Ca²⁺/Na⁺ ratio by 69.7 and 79.7% in 2023/2024 and 2024/2025, respectively, while lowering Na⁺ by 30.6 and 32.0%. Importantly, the CA × CU-PGPR interaction exerted a synergistic effect, as CA_200_ combined with *B. spizizenii* resulted in the highest increase in leaf N (45.8 and 30.4%), K⁺ (67.4 and 35.3%), Ca²⁺ (50.9 and 80.7%), while simultaneously recording the greatest decline in Na⁺ (48.8 and 42.9%) in 2023/2024 and 2024/2025, respectively. These effects translated into remarkable enhancements in ion balance, with K⁺/Na⁺ ratios rising by 234.3 and 134.3% and Ca²⁺/Na⁺ ratios by 199.2 and 178.3% in 2023/2024 and 2024/2025, respectively, compared with CA_0_ × NI.

**Table 6:** Effect of citric acid (CA) soil application rate, inoculation with citrate-utilizing plant growth-promoting rhizobacteria (CU-PGPR) strains, and their interaction on nitrogen (N), phosphorus (P), and potassium (K^+^) contents of the leaf blade and petiole of* Vitis vinifera* L. cv. ‘Superior Seedless’ grown under arid saline calcareous soil conditions during 2023/2024 and 2024/2025 seasons

Factor		Leaf *N* (%)		Leaf *P* (%)		Leaf K^+^ (%)	
		2023/2024	2024/2025	2023/2024	2024/2025	2023/2024	2024/2025
CA							
CA_0_		1.34 ± 0.08c	1.39 ± 0.02c	0.24 ± 0.01a	0.24 ± 0.01b	0.47 ± 0.04b	0.59 ± 0.02b
CA_100_		1.48 ± 0.03b	1.54 ± 0.06b	0.25 ± 0.00a	0.27 ± 0.01a	0.59 ± 0.02a	0.68 ± 0.02a
CA_200_		1.61 ± 0.08a	1.72 ± 0.03a	0.26 ± 0.01a	0.28 ± 0.01a	0.61 ± 0.03a	0.67 ± 0.03a
CU-PGPR							
NI		1.31 ± 0.05c	1.47 ± 0.06c	0.23 ± 0.01c	0.26 ± 0.01b	0.48 ± 0.02c	0.62 ± 0.03b
* H. marinus*		1.47 ± 0.05b	1.56 ± 0.05b	0.27 ± 0.01a	0.28 ± 0.01a	0.56 ± 0.02b	0.65 ± 0.02a
* B. spizizenii*		1.65 ± 0.06a	1.63 ± 0.05a	0.25 ± 0.01b	0.26 ± 0.01b	0.64 ± 0.04a	0.67 ± 0.02a
CA × CU-PGPR							
CA_0_	NI	1.20 ± 0.05f	1.35 ± 0.06f	0.21 ± 0.01e	0.23 ± 0.01d	0.43 ± 0.02 g	0.51 ± 0.02e
	*H. marinus*	1.35 ± 0.07d	1.41 ± 0.00e	0.25 ± 0.01 cd	0.25 ± 0.01c	0.50 ± 0.02f	0.63 ± 0.02d
	*B. spizizenii*	1.48 ± 0.12c	1.43 ± 0.04e	0.25 ± 0.01 cd	0.24 ± 0.01d	0.50 ± 0.04f	0.64 ± 0.03 cd
CA_100_	NI	1.26 ± 0.04e	1.41 ± 0.06e	0.24 ± 0.01d	0.27 ± 0.01b	0.47 ± 0.01f	0.71 ± 0.03a
	*H. marinus*	1.46 ± 0.08c	1.50 ± 0.13d	0.26 ± 0.01b	0.29 ± 0.01a	0.60 ± 0.05c	0.67 ± 0.04a-d
	*B. spizizenii*	1.71 ± 0.05a	1.70 ± 0.03b	0.25 ± 0.01b-d	0.26 ± 0.01bc	0.69 ± 0.01b	0.68 ± 0.05a-d
CA_200_	NI	1.47 ± 0.01c	1.64 ± 0.07c	0.24 ± 0.01d	0.27 ± 0.01b	0.54 ± 0.01e	0.66 ± 0.05b-d
	*H. marinus*	1.60 ± 0.02b	1.76 ± 0.05a	0.28 ± 0.01a	0.29 ± 0.01a	0.57 ± 0.02d	0.66 ± 0.05a-d
	*B. spizizenii*	1.75 ± 0.14a	1.76 ± 0.06a	0.26 ± 0.01b	0.27 ± 0.01b	0.72 ± 0.09a	0.69 ± 0.05ab
Significance							
CA		0.0001^**^	0.0002^**^	0.0775^*^	0.0014^*^	0.0031^*^	0.0183^*^
CU-PGPR		< 0.0001^**^	< 0.0001^**^	< 0.0001^**^	0.0001^**^	< 0.0001^**^	0.0038^*^
CA × CU-PGPR		0.0034^**^	< 0.0001^**^	0.0242^*^	0.0267^*^	< 0.0001^**^	0.0004^*^

Values are presented as means ± standard error (*n* = 3). Means followed by the same letter are not significantly different at *p* > 0.05 according to Duncan’s multiple range test. Asterisks indicate levels of significance (^*^p ≤ 0.05 and ^**^p ≤ 0.01)

*CA*_0_ non-CA-treated vines, *CA*_100_ CA at 100 g vine^− 1^, *CA*_200_ CA at 200 g vine^− 1^, *NI* non-CU-PGPR-inoculated vines, *H. marinus*= inoculation with LQ3S3EG isolate, *B. spizizenii*= inoculation with LQ1S14EG isolate

**Table 7:** Effect of citric acid (CA) soil application rate, inoculation with citrate-utilizing plant growth-promoting rhizobacteria (CU-PGPR) strains, and their interaction on calcium (Ca^2+^) and sodium (Na^+^) content of potassium (K^+^)/Na^+^ and Ca^2+^/Na^+^ ratios of the leaf blade and petiole of *Vitis vinifera* L. cv. ‘Superior Seedless’ grown under arid saline calcareous soil conditions during 2023/2024 and 2024/2025 seasons

Factor		Leaf Ca^2+^ (%)		Leaf Na^+^ (%)		Leaf Ca^2+^/Na^+^ ratio	
		2023/2024	2024/2025	2023/2024	2024/2025	2023/2024	2024/2025
CA							
CA_0_		0.61 ± 0.03c	0.73 ± 0.08c	0.33 ± 0.03a	0.23 ± 0.02a	1.95 ± 0.29c	3.13 ± 0.37c
CA_100_		0.67 ± 0.03b	0.84 ± 0.04b	0.29 ± 0.02b	0.21 ± 0.02b	2.36 ± 0.09b	4.07 ± 0.36b
CA_200_		0.73 ± 0.02a	0.93 ± 0.04a	0.28 ± 0.03b	0.19 ± 0.02c	2.76 ± 0.32a	4.77 ± 0.34a
CU-PGPR							
NI		0.62 ± 0.03b	0.69 ± 0.05b	0.36 ± 0.02a	0.25 ± 0.02a	1.75 ± 0.16c	2.81 ± 0.28c
* H. marinus*		0.70 ± 0.04a	0.93 ± 0.03a	0.29 ± 0.02ab	0.20 ± 0.02b	2.35 ± 0.16ab	4.10 ± 0.31b
* B. spizizenii*		0.69 ± 0.04a	0.90 ± 0.04a	0.25 ± 0.03c	0.17 ± 0.02c	2.97 ± 0.33a	5.05 ± 0.46a
CA × CU-PGPR							
CA_0_	NI	0.53 ± 0.03e	0.57 ± 0.07d	0.41 ± 0.02a	0.28 ± 0.01a	1.30 ± 0.07e	2.12 ± 0.11 h
	*H. marinus*	0.63 ± 0.03 cd	0.88 ± 0.06b	0.31 ± 0.07d	0.22 ± 0.05bc	2.04 ± 0.17d	2.86 ± 0.29 g
	*B. spizizenii*	0.67 ± 0.03c	0.77 ± 0.03c	0.27 ± 0.06e	0.17 ± 0.03d	2.51 ± 0.21bc	4.42 ± 0.32d
CA_100_	NI	0.67 ± 0.03c	0.73 ± 0.08c	0.35 ± 0.03b	0.24 ± 0.04b	1.91 ± 0.12d	3.23 ± 0.34f
	*H. marinus*	0.73 ± 0.03b	0.90 ± 0.06b	0.28 ± 0.03e	0.21 ± 0.04 cd	2.50 ± 0.15bc	4.14 ± 0.15e
	*B. spizizenii*	0.60 ± 0.04d	0.90 ± 0.06b	0.24 ± 0.02f	0.17 ± 0.01ef	2.67 ± 0.12b	4.83 ± 0.39c
CA_200_	NI	0.67 ± 0.03c	0.73 ± 0.08c	0.33 ± 0.03c	0.24 ± 0.04b	2.04 ± 0.12d	3.10 ± 0.23f
	*H. marinus*	0.73 ± 0.03b	1.03 ± 0.06a	0.31 ± 0.04d	0.18 ± 0.01d-f	2.36 ± 0.22c	5.31 ± 0.43b
	*B. spizizenii*	0.80 ± 0.03a	1.03 ± 0.03a	0.21 ± 0.07 g	0.16 ± 0.01f	3.89 ± 0.36a	5.90 ± 0.31a
Significance							
CA		0.0009^*^	0.0004^*^	0 0012^*^	< 0.0001^**^	0.0001^**^	< 0.0001^**^
CU-PGPR		0.0005^*^	< 0.0001^**^	< 0.0001^**^	< 0.0001^**^	< 0.0001^**^	< 0.0001^**^
CA × CU-PGPR		0.0004^*^	0.0214^*^	< 0.0001^**^	0.0401^*^	< 0.0001^**^	< 0.0001^**^

Values are presented as means ± standard error (*n* = 3). Means followed by the same letter are not significantly different at *p* > 0.05 according to Duncan’s multiple range test. Asterisks indicate levels of significance (^*^p ≤ 0.05 and ^**^p ≤ 0.01)

*CA*_0_ non-CA-treated vines, *CA*_100_ CA at 100 g vine^− 1^, *CA*_200_ CA at 200 g vine^− 1^, *NI* non-CU-PGPR-inoculated vines, *H. marinus*= inoculation with LQ3S3EG isolate, *B. spizizenii*= inoculation with LQ1S14EG isolate

Soil application of CA significantly improved the leaf and petiole contents of Fe²⁺, Zn²⁺, and Mn²⁺ in grapevines during the 2023/2024 and 2024/2025 growing seasons (Table 8). Compared with the CA_0_ vines control, the CA_200_ application resulted in marked increases in Fe²⁺ by 65.2 and 64.3%, Zn²⁺ by 58.6 and 59.5%, and Mn²⁺ by 50.7 and 60.2%, respectively. Similarly, inoculation with CU-PGPR substantially enhanced leaf and petiole micronutrients, with the *B. spizizenii* strain exhibiting superior performance over both *H. marinus* and the NI control. Relative to NI control, *B. spizizenii* increased Fe²⁺ by 25.5% in both seasons, Zn²⁺ by 40.5 and 43.6%, and Mn²⁺ by 50.6 and 39.0% in the 2023/2024 and 2024/2025 seasons, respectively. Notably, the CA × CU-PGPR interaction exerted the most synergistic effect, particularly under the CA_200_ × *B. spizizenii* interaction, which recorded the greatest contents of Fe²⁺ (202.90 and 204.9 mg kg⁻¹) and Zn²⁺ (89.6 and 90.4 mg kg⁻¹) during the 2023/2024 and 2024/2025 growing seasons, respectively, and Mn²⁺ (170.0 mg kg⁻¹ in both seasons). These values represented substantial enhancements of 83.3 and 82.8%, 113.3 and 121.6%, and 136.8 and 132.2% for Fe²⁺, Zn²⁺, and Mn²⁺ for the 2023/2024 and 2024/2025 growing seasons, respectively, when compared to the CA_0_ × NI control.

**Table 8:** Effect of citric acid (CA) soil application rate, inoculation with citrate-utilizing plant growth-promoting rhizobacteria (CU-PGPR) strains, and their interaction on iron (Fe^+2^), zinc (Zn^+2^), and manganese (Mn^+2^) contents of the leaf blade and petiole of *Vitis vinifera* L. cv. ‘Superior Seedless’ grown under arid saline calcareous soil conditions during 2023/2024 and 2024/2025 seasons

Factor		Leaf Fe^+ 2^ (mg kg^− 1^ DrW)		Leaf Zn^+ 2^ (mg kg^− 1^ DrW)		Leaf Mn^+ 2^ (mg kg^− 1^ DrW)	
		2023/2024	2024/2025	2023/2024	2024/2025	2023/2024	2024/2025
CA							
CA_0_		112.47 ± 0.2c	113.43 ± 0.34c	43.10 ± 3.11c	42.47 ± 3.3c	101.40 ± 5.8c	95.73 ± 5.6c
CA_100_		144.50 ± 2.6b	145.27 ± 2.54b	52.10 ± 0.85b	52.47 ± 3.8b	121.73 ± 5.4b	114.27 ± 5.4b
CA_200_		185.77 ± 4.6a	186.37 ± 4.56a	68.37 ± 5.62a	67.73 ± 6.0a	152.77 ± 7.0a	153.33 ± 7.1a
CU-PGPR							
NI		128.83 ± 8.2c	129.63 ± 8.01c	46.53 ± 1.47c	45.67 ± 1.25c	99.00 ± 7.7c	99.57 ± 7.9c
* H. marinus*		152.20 ± 1.6a	152.73 ± 1.34a	51.67 ± 5.20a	51.43 ± 7.45a	127.77 ± 5.5a	125.33 ± 9.6a
* B. spizizenii*		161.70 ± 3.0a	162.70 ± 3.21a	65.37 ± 3.39a	65.57 ± 3.41a	149.13 ± 9.4a	138.43 ± 5.5a
CA × CU-PGPR							
CA_0_	NI	110.70 ± 0.13i	112.10 ± 2.55 g	42.00 ± 2.25 g	40.80 ± 2.03 h	71.80 ± 3.6 h	73.20 ± 4.1 h
	*H. marinus*	113.00 ± 0.06 h	114.10 ± 3.26f	42.60 ± 2.17 g	42.70 ± 2.01 g	100.00 ± 4.1 g	101.0 ± 3.7f
	*B. spizizenii*	113.70 ± 0.01d	114.10 ± 2.03f	44.70 ± 2.88f	43.90 ± 2.56f	132.40 ± 5.6d	113.0 ± 4.1 C
CA_100_	NI	114.50 ± 0.20f	115.20 ± 2.98f	46.00 ± 2.12f	47.20 ± 2.29e	100.20 ± 4.1 g	100.50 ± 4.2 g
	*H. marinus*	150.50 ± 0.07e	151.50 ± 3.29e	48.50 ± 2.09e	47.80 ± 2.09e	120.00 ± 6.0f	110.00 ± 4.8 g
	*B. spizizenii*	168.50 ± 0.09c	169.10 ± 3.14c	61.80 ± 2.03c	62.40 ± 2.07c	145.00 ± 6.0c	132.30 ± 5.3d
CA_200_	NI	161.30 ± 0.04d	161.60 ± 4.17d	51.60 ± 2.70d	49.00 ± 2.45d	125.00 ± 6.1e	125.00 ± 5.6e
	*H. marinus*	193.10 ± 0.39b	192.60 ± 4.33b	63.90 ± 2.19b	63.80 ± 2.06b	163.30 ± 6.7b	165.00 ± 5.6b
	*B. spizizenii*	202.90 ± 0.19a	204.90 ± 5.95a	89.60 ± 2.23a	90.40 ± 2.03a	170.00 ± 6.0a	170.00 ± 6.7a
Significance							
CA		< 0.0001^**^	< 0.0001^**^	< 0.0001^**^	< 0.0001^**^	< 0.0001^**^	< 0.0001^**^
CU-PGPR		< 0.0001^**^	< 0.0001^**^	< 0.0001^**^	< 0.0001^**^	< 0.0001^**^	< 0.0001^**^
CA × CU-PGPR		< 0.0001^**^	< 0.0001^**^	< 0.0001^**^	< 0.0001^**^	< 0.0001^**^	< 0.0001^**^

Values are presented as means ± standard error (*n* = 3). Means followed by the same letter are not significantly different at *p* > 0.05 according to Duncan’s multiple range test. Asterisks denote statistical significance (** *p* ≤ 0.01)

*CA*_0_ non-CA-treated vines, *CA*_100_ CA at 100 g vine^-1^, *CA*_200_ CA at 200 g vine-1, *NI* non-CU-PGPR-inoculated vines, *H. marinus* inoculation with LQ3S3EG isolate, *B. spizizenii* inoculation with LQ1S14EG isolate

### Physio-biochemical responses

Soil CA application significantly enhanced the VRWC of *V. vinifera* L. cv. ‘Superior Seedless’ under arid saline calcareous stress conditions; however, its effect on MSI remained statistically non-significant (Table 9). Compared to the CA_0_, soil CA_200_ application increased VRWC by 16.4 and 10.0% during the 2023/2024 and 2024/2025 seasons, respectively. Similarly, rhizospheric inoculation with CU-PGPR significantly improved salt-stressed grapevine water status. Among the tested bacterial strains, *B. spizizenii* was the most effective, enhancing MSI by 11.6 and 26.0%, and VRWC by 13.1 and 6.8% over the NI control vines during the 2023/2024 and 2024/2025 seasons, respectively. Notably, the CA × CU-PGPR interaction exerted synergistic effects on VRWC, with the CA_200_ × *H. marinus* and CA_200_ × *B. spizizenii* combinations exhibiting complementary effects on VRWC, significantly exceeding the CA_0_ × NI control by 33.0 and 30.8% in the 2023/2024 season, and 15.4 and 14.6% in the 2024/2025 season, respectively.

**Table 9:** Effect of citric acid (CA) soil application rate, inoculation with citrate-utilizing plant growth-promoting rhizobacteria (CU-PGPR) strains, and their interaction on membrane stability index (MSI), vine relative water content (RWC), free proline content (ProC) and total phenolic (TPh) content of *Vitis vinifera* L. cv. ‘Superior Seedless’ grown under arid saline calcareous soil conditions during 2023/2024 and 2024/2025 seasons

Factor		MSI (%)		VRWC (%)		Free ProC (µg g^− 1^ DrW)	
		2023/2024	2024/2025	2023/2024	2024/2025	2023/2024	2024/2025
CA							
CA_0_		36.65 ± 1.49	49.37 ± 1.82	60.73 ± 1.89c	66.00 ± 1.41c	1.28 ± 0.09c	1.40 ± 0.07b
CA_100_		40.81 ± 0.92	51.66 ± 1.87	67.32 ± 1.31b	70.12 ± 1.40b	1.50 ± 0.12b	1.49 ± 0.09a
CA_200_		44.19 ± 1.42	52.49 ± 3.11	70.69 ± 0.84a	72.63 ± 1.02a	1.63 ± 0.09a	1.42 ± 0.07b
CU-PGPR							
NI		38.53 ± 1.60b	46.28 ± 0.64c	61.65 ± 1.97c	66.61 ± 1.67b	1.38 ± 0.11c	1.29 ± 0.06c
* H. marinus*		40.12 ± 1.47b	48.92 ± 2.57b	67.36 ± 1.76b	71.00 ± 1.02a	1.58 ± 0.06a	1.45 ± 0.11b
* B. spizizenii*		43.00 ± 1.13a	58.33 ± 2.16a	69.72 ± 0.80a	71.14 ± 1.00a	1.44 ± 0.11b	1.57 ± 0.06a
CA × CU-PGPR							
CA_0_	NI	34.19 ± 1.85	45.51 ± 1.03	54.62 ± 1.00f	63.97 ± 0.58c	1.23 ± 0.07d	1.27 ± 0.05d
	*H. marinus*	36.31 ± 0.57	46.49 ± 0.51	60.11 ± 0.09e	66.58 ± 0.23bc	1.39 ± 0.05c	1.45 ± 0.15b
	*B. spizizenii*	39.46 ± 1.95	56.10 ± 1.59	67.47 ± 0.31c	67.45 ± 1.63b	1.21 ± 0.02d	1.49 ± 0.05b
CA_100_	NI	39.15 ± 0.62	47.53 ± 1.28	62.36 ± 0.69de	65.09 ± 2.06bc	1.34 ± 0.05d	1.25 ± 0.04d
	*H. marinus*	39.82 ± 1.65	43.74 ± 1.34	69.36 ± 0.99ab	72.63 ± 0.23a	1.73 ± 0.06a	1.57 ± 0.05a
	*B. spizizenii*	43.45 ± 1.40	63.72 ± 2.29	70.23 ± 0.29ab	72.65 ± 0.31a	1.42 ± 0.08c	1.64 ± 0.06a
CA_200_	NI	42.26 ± 1.31	45.80 ± 0.98	67.98 ± 0.40bc	70.77 ± 0.34a	1.57 ± 0.03b	1.36 ± 0.03c
	*H. marinus*	44.22 ± 0.45	56.52 ± 1.43	72.62 ± 1.29a	73.80 ± 0.34a	1.62 ± 0.13b	1.32 ± 0.09 cd
	*B. spizizenii*	46.10 ± 0.81	55.16 ± 0.84	71.47 ± 0.90a	73.33 ± 0.03a	1.70 ± 0.13a	1.58 ± 0.08a
Significance							
CA		< 0.2642^ns^	< 0.1097^ns^	< 0.0001^**^	< 0.0147^**^	< 0.0001^**^	< 0.0095^*^
CU-PGPR		< 0.0280^*^	< 0.0125^*^	< 0.0001^**^	< 0.0001^**^	< 0.0001^**^	< 0.0451^*^
CA × CU-PGPR		< 0.9640^ns^	< 0.0536^ns^	< 0.0001^**^	< 0.0001^**^	< 0.0001^**^	< 0.0003^**^

Values are presented as means ± standard error (*n* = 3). Means followed by the same letter are not significantly different at *p* > 0.05 according to Duncan’s multiple range test. Asterisks denote statistical significance (^**^p ≤ 0.01) and “ns” denotes a non-significant difference (*p* > 0.05). DrW = dry weight

*CA*_0_ non-CA-treated vines, *CA*_100_ CA at 100 g vine^− 1^, *CA*_200_ CA at 200 g vine^− 1^g/vine, *NI* non-CU-PGPR-inoculated vines, *H. marinus*= inoculation with LQ3S3EG isolate, *B. spizizenii*= inoculation with LQ1S14EG isolate

The individual application of CA significantly enhanced both free ProC and TPh content *V. vinifera* L. cv. ‘Superior Seedless’ under arid saline calcareous soil conditions in both growing seasons (Tables 9 and Fig. 2b). The CA_200_ resulted in the highest free ProC, with increases of 27.3 and 1.4% over the CA_0_-treated vines in 2023/2024 and 2024/2025, respectively, and improved TPh content by 14.9 and 20.3%, respectively. Regarding the CU-PGPR effect, both *H. marinus* and *B. spizizenii* strains significantly elevated free ProC and TPh content compared to the NI control. In particular, *B. spizizenii* increased free ProC by 4.3 and 21.7% and TPh content by 10.3 and 18.3% over NI in the respective seasons. The CA × CU-PGPR interaction revealed a synergistic enhancement, with the CA_200_ × *B. spizizenii* combination being the most effective, elevating free ProC by 38.2 and 24.4% and TPh content by 60.4 and 90.5% compared to the CA_0_ × NI control in 2023/2024 and 2024/2025, respectively.

### Grape yield and its quality traits

The application of CA significantly improved yield-expressed traits, pruning WW and grape yield of ‘Superior Seedles’ vines over the 2023/2024 and 2024/2025 growing seasons (Tables 10 and Fig. 2c). Relative to the CA_0_ vines control, the CA_200_ level increased pruning WW by 33.3 and 46.2%, and grape yield by 57.3 and 44.0% for the 2023/2024 and 2024/2025 growing seasons, respectively. In parallel, inoculation with CU-PGPR elicited a significant positive impact on vine performance. Specifically, *B. spizizenii* markedly outperformed the NI control, increasing pruning WW by 27.3 and 39.6%, and enhancing grape yield by 29.1 and 37.6% during the 2023/2024 and 2024/2025 seasons, respectively. A significant CA × CU-PGPR interaction was observed, wherein the CA_200_ × *B. spizizenii* interaction application resulted in the most substantial improvements-raising pruning WW by 66.2 and 95.8%, and grape yield by 97.6 and 96.3% compared to the CA_0_ × NI control across the respective growing seasons.

**Table 10:** Effect of citric acid (CA) soil application rate, inoculation with citrate-utilizing plant growth-promoting rhizobacteria (CU-PGPR) strains, and their interaction on pruning wood weight (WW), grapes yield, fruit total soluble solids/acidity (TSS/AC) ratio, fruit firmness of *Vitis vinifera* L. cv. ‘Superior Seedless’ grown under arid saline calcareous soil conditions during 2023/2024 and 2024/2025 seasons

Factor		Pruning WW (kg vine^− 1^)		Fruit TSS/AC ratio		Fruit firmness (kg cm^− 2^)	
		2023/2024	2024/2025	2023/2024	2024/2025	2023/2024	2024/2025
CA							
CA_0_		0.87 ± 0.07c	0.91 ± 0.09b	35.5 ± 1.62c	33.1 ± 1.77c	1.21 ± 0.03b	1.20 ± 0.04b
CA_100_		1.03 ± 0.06b	1.14 ± 0.09a	44.5 ± 1.79b	43.5 ± 1.80b	1.30 ± 0.03a	1.34 ± 0.03a
CA_200_		1.16 ± 0.01a	1.33 ± 0.04a	49.1 ± 2.12a	50.2 ± 3.52a	1.34 ± 0.03a	1.39 ± 0.04a
CU-PGPR							
NI		0.88 ± 0.06b	0.91 ± 0.11b	37.3 ± 2.42c	37.1 ± 3.58c	1.23 ± 0.03b	1.24 ± 0.04c
* H. marinus*		1.06 ± 0.05a	1.21 ± 0.06a	43.5 ± 2.32b	42.3 ± 3.52b	1.29 ± 0.03a	1.31 ± 0.05b
* B. spizizenii*		1.12 ± 0.04a	1.27 ± 0.05a	48.4 ± 3.62a	47.5 ± 3.72a	1.32 ± 0.04a	1.38 ± 0.04a
CA × CU-PGPR							
CA_0_	NI	0.71 ± 0.04b	0.71 ± 0.01e	29.3 ± 1.02	28.3 ± 1.17	1.16 ± 0.04	1.13 ± 0.04
	*H. marinus*	0.87 ± 0.03b	0.97 ± 0.04d	36.6 ± 1.55	33.8 ± 1.18	1.21 ± 0.03	1.21 ± 0.04
	*B. spizizenii*	1.03 ± 0.09a	1.06 ± 0.04 cd	40.7 ± 1.02	37.3 ± 1.34	1.25 ± 0.03	1.25 ± 0.04
CA_100_	NI	0.82 ± 0.03b	0.77 ± 0.00e	40.5 ± 1.69	39.5 ± 1.62	1.26 ± 0.02	1.26 ± 0.04
	*H. marinus*	1.12 ± 0.02a	1.32 ± 0.01a	45.4 ± 1.82	43.6 ± 1.89	1.33 ± 0.03	1.30 ± 0.04
	*B. spizizenii*	1.16 ± 0.08a	1.35 ± 0.08a	47.7 ± 1.52	47.5 ± 2.82	1.31 ± 0.04	1.44 ± 0.05
CA_200_	NI	1.12 ± 0.01a	1.25 ± 0.07ab	42.0 ± 1.55	43.5 ± 2.55	1.28 ± 0.04	1.31 ± 0.04
	*H. marinus*	1.18 ± 0.03a	1.36 ± 0.04a	48.3 ± 2.52	49.4 ± 2.74	1.33 ± 0.04	1.42 ± 0.05
	*B. spizizenii*	1.18 ± 0.01a	1.39 ± 0.05a	56.9 ± 2.52	57.8 ± 2.82	1.40 ± 0.04	1.44 ± 0.05
Significance							
CA		< 0.0023^*^	< 0.0018^*^	0.0008^**^	0.0003^**^	0.0066^*^	0.0026^**^
CU-PGPR		< 0.0002^*^	< 0.0001^**^	0.0008^**^	<0.0001^**^	0.0002^**^	<0.0001^**^
CA × CU-PGPR		< 0.0414^*^	< 0.0017^*^	0.6298^ns^	0.2137^ns^	0.1056^ns^	0.1482^ns^

Values are presented as means ± standard error (*n* = 3). Means followed by the same letter are not significantly different at *p* > 0.05 according to Duncan’s multiple range test. Asterisks denote statistical significance (^**^p ≤ 0.01) and “ns” denotes a non-significant difference (*p* > 0.05)

*CA*_0_ non-CA-treated vines, *CA*_100_ CA at 100 g vine^− 1^, *CA*_200_ CA at 200 g vine^− 1^, *NI* non-CU-PGPR-inoculated vines, *H. marinus*= inoculation with LQ3S3EG isolate, *B. spizizenii*= inoculation with LQ1S14EG isolate

The results revealed a substantial improvement in fruit TSS/AC ratio and firmness in response to soil CA application, CU-PGPR inoculation, and their interaction. Increasing CA rate from 0 to 200 g vine⁻¹ enhanced the TSS/AC ratio by 38.3 and 51.7%, and fruit firmness by 10.7 and 15.8%, during the 2023/2024 and 2024/2025 seasons, respectively, relative to CA_0_. Similarly, CU-PGPR inoculation markedly elevated both parameters, with *B. spizizenii* outperforming other treatments, inducing increases of 29.8 and 28.0% in TSS/AC ratio, and 7.3 and 11.3% in firmness compared to the NI control during the 2023/2024 and 2024/2025 seasons, respectively. While the CA × CU-PGPR interaction showed no statistically significant effects (*p* ≤ 0.05), notable numerical enhancements were observed with the combined application of CA_200_ and *B. spizizenii*, recording the highest TSS/AC ratio (56.9 and 57.8) and fruit firmness (1.40 and 1.44 kg cm^-2^) across the two seasons, representing 94.2 and 104.3% increases in TSS/AC ratio, and 20.7 and 27.4% increases in firmness, respectively, over the CA_0_ × NI interaction.

### Multivariate principal component (PCA) and correlation analyses

**Fig. 3:**
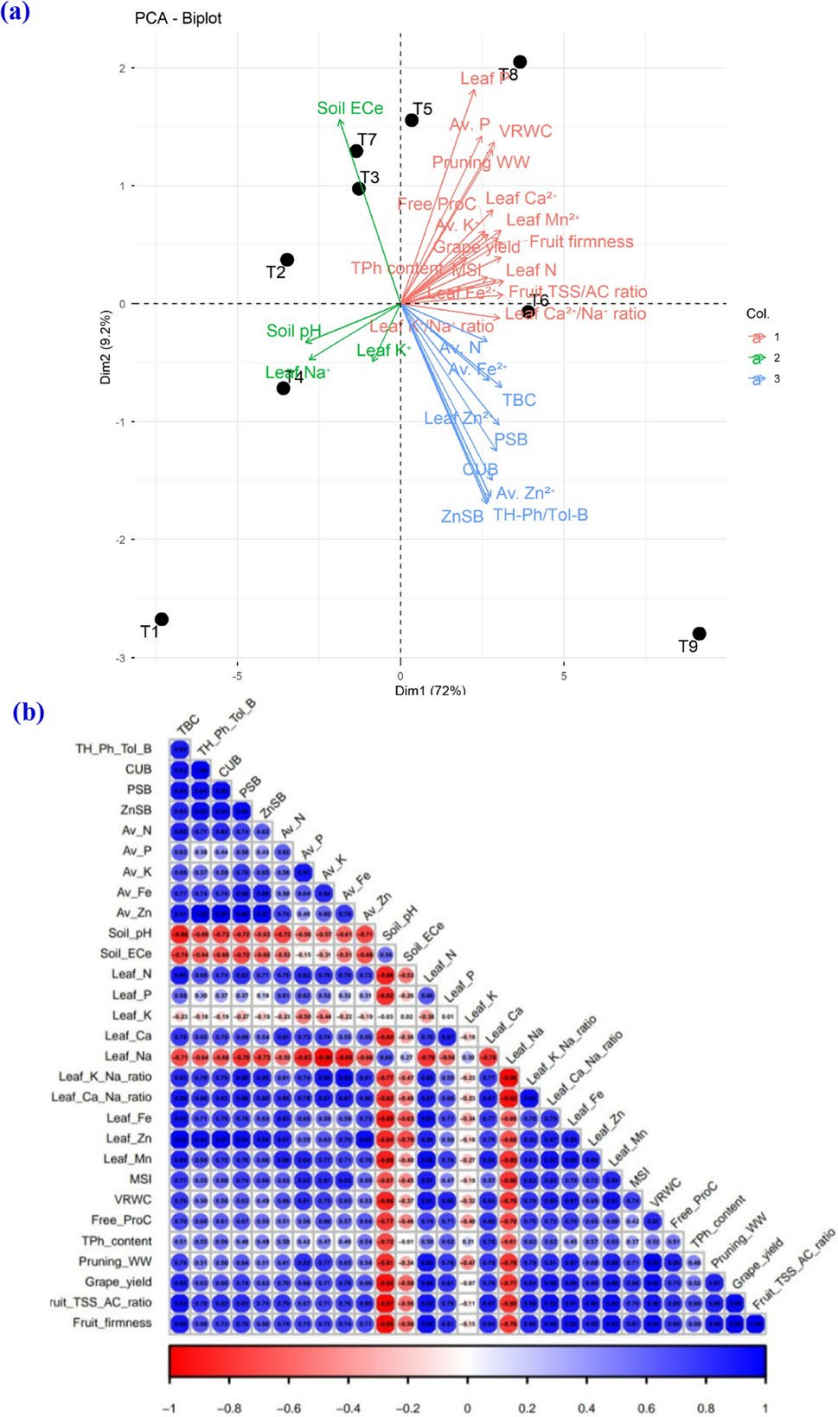
Multivariate assessment of CA × CU-PGPR treatment effects through (**a**) PCA ordination of microbial–soil–plant traits and (**b**) correlogram correlation matrix of microbial–soil–plant traits in *V. vinifera* cv. ‘Superior Seedless’ grown under saline–calcareous conditions. Circle hue represents correlation direction and strength, while circle’s size and significance level correspond. (*) refers to a significant (*p* ≤ 0.05) correlation. T1: CA_0_ × NI, T2: CA_0_ × *H. marinus*, T3: CA_0_ × *B. spizizenii*, T4: CA_100_ × NI, T5: CA_100_ × *H. marinus*, T6: CA_100_ × *B. spizizenii*, T7: CA_200_ × NI, T8: CA_200_ × *H. marinus*, and T9: CA_200_ × *B. spizizenii.* TBC: total bacteria count, TH-Ph/Tol-B: total halophilic and halotolerant bacteria, CUB: citrate-utilizing bacteria, PSB: phosphate-solubilizing bacteria, ZnSB: zinc-solubilizing bacteria, Av. N, Av. P, Av. K⁺, Av. Fe^+2^, Av. Zn^+2^= available nitrogen, phosphorus, potassium, iron, and zinc, ECe= electrical conductivity for soil past extract, Leaf N, Leaf P, Leaf K⁺, Leaf Ca²⁺, Leaf Na⁺, Leaf Fe^+2^, Leaf Zn^+2^, and Leaf Mn^+2^=Leaf nitrogen, phosphorus, potassium, calcium, sodium, iron, zinc, and manganese, MSI: membrane stability index, VRWC: vine relative water content, Free ProC: free proline content, TPh content: total phenolic content, Pruning WW: pruning wood weight, and Fruit TSS/AC ratio= Fruit total soluble solids/acidity ratio. Col.: color. Red vectors (Group 1) represent plant physiological traits, leaf nutritional status, and yield components; green vectors (Group 2) indicate soil salinity, soil reaction (pH), and associated stress ions, and blue vectors (Group 3) denote rhizospheric microbial populations and available soil nutrients. Each black dot in sub-figure (A) refers to a treatment number. Values based on average of 2023/2024 and 2024/2025 seasons

The PCA biplot (Fig. 3a) distinctly separated the studied treatments in multidimensional space, emphasizing the integrative influence of CA and CU-PGPR co-application on grapevine adaptability under saline-calcareous conditions. The 1st two components (Dim1 = 72% and Dim2 = 9.2%) jointly explained > 81% of the total variability. The T9 (CA_200_ × *B. spizizenii*) was distinctly positioned on the positive Dim1, closely associated with improved leaf nutrition (N, P, K⁺, Ca²⁺, Fe²⁺, Zn²⁺, and Mn²⁺), ionic homeostasis (K⁺/Na⁺ and Ca²⁺/Na⁺ ratios), physiological (MSI, VRWC, free ProC, and TPh content), grape yield, and quality traits (TSS/AC ratio and firmness). Conversely, T1 (CA_0_ × NI as a control), T2 (CA₀ × *H. marinus*), and T3 (CA_0_ × *B. spizizenii*) treatments clustered toward the negative side of Dim1, correlating with higher soil pH, EC_e_, and leaf Na⁺, indicating poor rhizospheric conditions. The blue microbial and nutrient vector cluster (TBC, PSB, CUB, ZnSB, TH-Ph/Tol-B, Av. N, Av. Fe²⁺, Av. Zn²⁺, and Leaf Zn²⁺) aligned positively with Dim1, signifying that enhanced microbial activity positively contributed to nutrient solubilization. Meanwhile, soil pH and EC_e_ vectors opposed this cluster, indicating their antagonistic effect on grapevine productivity and it quality. Collectively, the PCA confirms that the synergistic CA × CU-PGPR interaction, particularly CA_200_ × *B. spizizenii*, as a driver of rhizospheric soil amelioration, microbial functionality, and physiological resilience under saline-calcareous stress conditions.

The correlation heatmap (Fig. 3b) revealed an intricate interconnection among soil properties, leaf ionic balance, stress physiology, and horticultural performance, highlighting the integrative efficacy of the CA and CU-PGPR co-application. Strong positive interrelations were observed between key salinity tolerance indices (Leaf K⁺/Na⁺ and Ca²⁺/Na⁺ ratios, MSI, and VRWC), underscoring the functional importance of ionic homeostasis in cellular integrity under stress. Concurrently, these physiological benefits showed positive associations with soil microbial activity (CUB, PSB, and ZnSB) and the nutrients (Av. N, Av. P, Av. K⁺, Av. Fe²⁺, and Av. Zn²⁺) availability, suggesting a CU-PGPR-mediated enhancement of the rhizospheric functionality. Moreover, their positive linkages between these soil and grapevine parameters and ultimate yield and fruit TSS/AC ratio, emphasize the cascading influence of improved soil-plant relations to enhanced yield and fruit quality.

## Discussion

Soil salinity poses one of the most critical constraints to perennial fruit production, including *V. vinifera*, by triggering a cascade of structural and physio-biochemical disorders. It diminishes VRWC, destabilizes cellular membranes, and accelerates chlorophyll degradation, as reported in fruit crops [19, 83]. Salinity also promotes lipid peroxidation, suppresses photosynthesis, and enhances the accumulation of reactive oxygen species (ROS), thereby restricting root water flow [84, 85]. Moreover, ion toxicity and nutrient imbalances, especially Na⁺ excess, impair root meristem activity and disrupt ionic homeostasis in *V. vinifera* vines [1]. These cumulative effects suppress growth [86], reduce yields, and in severe cases lead to grapevine mortality [87]. Here, the present study investigated whether halotolerant and halophilic CU-PGPR, combined with CA, can mitigate these constraints. This occurs through modulation of the rhizosphere microbial community, enhanced nutrient availability, and improved grapevine resilience under saline–calcareous conditions. Based on their ability to adapt to salty environments, microbiota are differentiated into halotolerant or halophilic CU-PGPR. Halotolerant (i.e., salt-tolerant) strains can survive in media containing up to 25% NaCl or even in NaCl-free media entirely, whereas halophilic (i.e., salt-dependent) strains require salts for proliferation [26]. Halophilic bacteria are categorized based on their optimal salinity range for growth: mild halophiles (thrive at 1–3% NaCl), moderate halophiles (thrive at 3–15% NaCl), and extreme halophiles (thrive at 15–25% NaCl) [27].

Soil salinity is a key driver shaping the diversity and functionality of rhizosphere microbial communities. In this study, both inoculation with *B. spizizenii* or *H. marinus* and CA soil application significantly enhanced microbial counts in *V. vinifera* cv. ‘Superior Seedless’ vines compared with NI controls, with *B. spizizenii* consistently outperforming *H. marinus*. This difference reflects their ecological adaptations: *B. spizizenii*, being halotolerant, adapts efficiently to moderate salinity, whereas *H. marinus*, being moderately halophilic, requires higher salt levels for optimal proliferation. Such observations corroborate previous reports that halotolerant PGPR are generally more effective than halophiles in agricultural saline soils [88–90].

The stimulatory effect of CA is attributable to its twofold role as a C-rich chemoattractant and energetic substrate that selectively enriches citrate-metabolizing bacterial communities, particularly *Firmicutes* such as *Bacillus* [42, 48, 91]. Importantly, *B. spizizenii* inherently exhibits P- and Zn-solubilizing capabilities (Table 1), supporting its effective root colonization and functional performance under saline–calcareous conditions. Concurrently, CA application promoted biofilm formation and EPSs production, reinforcing microbial establishment under osmotic stress [92–94]. The resulting EPS matrix contributed to soil aggregation and partial Na⁺ immobilization, improving the rhizospheric microenvironment [31, 95–97]. Collectively, CA enriched citrate-utilizing bacteria, while the functional traits of *B. spizizenii* supported soil improvements. These effects enhanced total bacterial counts, including P- and Zn²⁺-solubilizers and Firmicutes. Consequently, these EPS processes fostered a biologically active rhizosphere that supports vine growth in saline calcareous soils.

Salinity stress-induced reductions in nutrient bioavailability are largely attributed to ionic competition, CO_3_^2−^ precipitation, and restricted microbial-mediated mineralization [98, 99]. The restructuring of microbial communities in response to treatments directly influenced rhizospheric nutrient dynamics. Through EPSs secretion, the inoculated strains contributed to the immobilization of toxic Na⁺ ions, coupled with better soil aggregation and water retention. Additionally, *B. spizizenii* or *H. marinus* exhibited P-solubilizing and siderophore-producing traits, thereby improving the nutrient (i.e., P, Fe²⁺, and Zn²⁺) bioavailability [100–102].

The synergistic interaction between CA supplementation and CU-PGPR inoculation mechanistically enhanced rhizospheric and plant resilience under saline-calcareous conditions. CA acted as a C-rich chemoattractant and acidifying agent, dissociating to release H⁺ ions, lowering soil pH, dissolving CaCO₃, and mobilizing immobilized P and micronutrients [1, 43, 49, 103]. This localized acidification improved cation-exchange processes and facilitated greater mobility of K⁺, Ca²⁺, and Mg²⁺ [104–106]. Concurrently, inoculated CU-PGPR strains, particularly *B. spizizenii*, secreted EPSs that reinforced biofilm formation, promoted soil aggregation, and partially immobilized Na⁺ ions, mitigating ionic toxicity [31, 93]. The EPS production also stabilized cytosolic pH and enhanced water retention, supporting root hydration and nutrient uptake [50, 107]. Together, these chemical and microbial processes regulated ionic homeostasis, improved nutrient bioavailability (P, Fe²⁺, and Zn²⁺), and maintained K⁺/Na⁺ and Ca²⁺/Na⁺ ratios favorable for physiological functions [18, 108–111]. The integration of CA-driven acidification and EPS-mediated soil responses created a biologically active rhizosphere that synergistically underpinned antioxidant defenses, photosynthetic efficiency, and overall vine performance in salt-affected soils [112].

Salinity stress is reported to compromise membrane integrity, reduce tissue water balance, and trigger ROS build-up in *V. vinifera* tissues [17]. Control vines exhibited pronounced decreases in MSI and VRWC, accompanied by cellular oxidative damage, supporting this concept. By contrast, CA × CU-PGPR treatments, particularly CA_200_ × *B. spizizenii*, significantly improved vine hydration status and antioxidative defense (Table 9). These effects can be attributed to CU-PGPR-mediated stimulation of free ProC biosynthesis and EPSs production, which stabilizes cytosolic pH, enhances osmotic adjustment, and supports root water uptake [91, 113, 114]. Concurrently, the CA application promoted osmolyte accumulation and enhanced H⁺ transport, thereby improving transmembrane water flux and stabilizing root integrity [47].

Moreover, the combined treatments markedly increased non-enzymatic antioxidants, particularly free ProC and TPh content. Proline acted dually as an osmoprotective and as a ROS-scavenging agent, while phenolic accumulation, likely via PGPR-induced phenylpropanoid pathway, contributed to reinforced cellular redox homeostasis [34, 115]. The CA also acted synergistically to stimulate free ProC biosynthesis and antioxidant activity [47, 116]. Together, these adjustments supported protective cellular responses, integrating ionic homeostasis with antioxidant defenses and maintaining functional photosynthetic machinery. This integrated stress-amelioration strategy conferred higher physiological endurance of *V. vinifera* exposed to salinity stress.

The reduction in crop productivity under salinity stress is generally associated with reduced carbohydrate partitioning, hormonal imbalance, hindered nutrient translocation, and oxidative damage [117, 118]. The non-CA or CU-PGPR-treated vines exhibited significant reductions in pruning DrW per vine and fruit yield, reflecting compromised photosynthesis, hormone signaling, and nutrient dynamics. However, CA × CU-PGPR applications nearly doubled productivity relative to controls (Table 10), while also enhancing fruit quality such as sugar accumulation, firmness, and the TSS/acid ratio [119–121].

Mechanistically, these yield and quality improvements arose from multiple synergistic processes. CU-PGPR-induced EPSs production may promote soil aggregation and water retention, thereby supporting better root resource acquisition [122, 123]. Concurrently, CA enhanced nutrient solubility and optimized ionic balance, collectively contributing to improved nutrient uptake and assimilation. These soil-plant interactions reinforced metabolic processes, maintained the vine’s canopy vigor, and sustained the allocation of photoassimilates to developing berries. Moreover, the fortification of antioxidant defenses minimized ROS-induced damage to photosynthetic structures, while ionic and biochemical adjustments preserved physiological resilience to salinity stress. Thus, the integrated pathway starts with microbial enrichment and CA-driven nutrient mobilization. This progresses through ionic regulation and antioxidant defense, ultimately restoring crop physiology. These interconnected mechanisms ultimately explain the substantial gains in *V. vinifera* productivity and commercially valuable fruit quality under saline-calcareous conditions. This evidence highlights the eco-agronomic value of integrating organic acid amendments with CU-PGPR as a sustainable approach for maintaining high yields in salt-affected viticulture systems.

Based on the synergistic mechanisms observed, this study practically recommends the soil application of citric acid at a rate of 200 g vine^− 1^ season^− 1^ (applied in four equal portions) combined primarily with *B. spizizenii* PGPR inoculation (8 L vine^− 1^ season^− 1^ in four splits of 2 L each). While *H. marinus* also contributes positively to rhizosphere improvement, its effect is comparatively smaller. This integrated amendment protocol offers growers an actionable framework to revitalize rhizosphere health, manage ionic balance, and sustain vine productivity under the constraints of arid saline-calcareous environments. The limitations of this investigation lie in its focus on a single *V. vinifera* cultivar ‘Superior Seedless’, two CU-PGPR strains (*B. spizizenii and H. marinus*), and a single organic acid. The experimental duration was relatively short, limited to two seasons and saline–calcareous soil conditions. Moreover, the study did not provide mechanistic insights at a molecular level, nor did it assess long-term soil-microbiome-plant interactions. Future research should integrate multi-omics approaches to uncover these mechanisms, test diverse PGPR–organic amendment synergies across different field conditions and grapevine cultivars, and conduct long-term assessments to evaluate microbial community stability and the sustainability of the observed benefits. Additionally, assessments of soil carbon dynamics, microbial survival, and cost-effectiveness would further strengthen the practical scalability and sustainability of this integrated bio-organic strategy.

## Conclusions

This study establishes that the synergistic co-application of citric acid and halophilic/halotolerant citrate-utilizing plant growth-promoting rhizobacteria, particularly *Bacillus spizizenii*, significantly enhances grapevine resilience in challenging saline-calcareous soils. Rather than acting in isolation, these combined treatments initiate a multi-tiered rhizosphere-plant mechanism. The process begins with foundational soil improvements, such as rhizosphere acidification and the stimulation of beneficial microbial communities, that effectively alleviate nutrient lock-up. In turn, these below-ground changes drive vital physio-biochemical adjustments within the plant, promoting ionic homeostasis, osmotic regulation, enhanced antioxidant defenses, and stabilized redox homeostasis. By restoring cellular integrity and revitalizing overall rhizosphere health, this integrative approach strengthens vine physiological performance to secure improved yield and fruit quality. Ultimately, integrating organic acid amendments with specialized plant growth-promoting rhizobacteria inoculants provides a vital, eco-efficient strategy to mitigate salinity stress in perennial fruit crops and revitalize degraded agricultural landscapes in arid regions. 

## Data Availability

The datasets used and/or analyzed during the current investigation are available from the corresponding author on reasonable request.

## Abbreviations

CA: Citric acid
PGPR: Plant growth-promoting rhizobacteria
CU-PGPR: Citrate-utilizing plant growth-promoting rhizobacteria
NI: Non-inoculated
EC_e_: Electrical conductivity of the soil extract
TBC: Total bacterial count
CUB: Citrate-utilizing bacteria
PSB: Phosphate-solubilizing bacteria
ZSB: Zinc-solubilizing bacteria
TH-Ph/Tol-B: Total halophilic and halotolerant bacteria
VRWC: Vine relative water content
MSI: Membrane stability index
TSS: Total soluble solids
RCBD: Randomized complete block design
IAA: Indole acetic acid
ACCD: 1-aminocyclopropane-1-carboxylate (ACC) deaminase
ProC: Proline content
TPh: Total phenolic
ROS: Reactive oxygen species
