# Correction: Integrative co-application of citric acid and halotolerant/halophilic citrate-utilizing PGPR enhances leaf ionic homeostasis, productivity, and quality of *Vitis vinifera* L. in saline-calcareous soils

**DOI:** 10.1186/s12870-026-09325-9

**Published:** 2026-06-26

**Authors:** Asmaa G. A. AbdelSamad, Ibrahim M. Ibrahim, Hamada R. Beheiry, Ahmed Shaaban, Hamdy A. Z. Hussein

**Affiliations:** 1 Horticulture Department, Faculty of Agriculture, Fayoum University, Fayoum, 63514 Egypt; 2 Qilu University of Technology (Shandong Academy of Sciences), Shandong Analysis and Test Center, Jinan, 250014 Shandong China; 3 Department of Agricultural Microbiology, Faculty of Agriculture, Fayoum University, Fayoum, 63514 Egypt; 4 Agronomy Department, Faculty of Agriculture, Fayoum University, Fayoum, 63514 Egypt

**Correction: BMC Plant Biol 26, 981 (2026)**

**https://doi.org/10.1186/s12870-026-09150-0**

Following publication of the original article [1], the authors found a scientific text error in the Abstract under Purpose section. Furthermore, the ORCID number of the second author, Ibrahim M. Ibrahim, has to be corrected. The specific details are given below:

**Abstract**: From “*Halomonas marinus*” should be corrected to “*Halobacillus marinus*”

**ORCID of Ibrahim M. Ibrahim**: From “0000-0002-5374-3508” should be corrected to “0000-0002-9221-1272”

The original articles [1] has been corrected.
